# Optimal Dosing and Timing of High-Dose Corticosteroid Therapy in Hospitalized Patients With COVID-19: Study Protocol for a Retrospective Observational Multicenter Study (SELECT)

**DOI:** 10.2196/48183

**Published:** 2023-06-02

**Authors:** Katrijn Daenen, Jilske A Huijben, Anders Boyd, Lieuwe D J Bos, Sara C M Stoof, Hugo van Willigen, Diederik A M P J Gommers, Hazra S Moeniralam, Corstiaan A den Uil, Nicole P Juffermans, Merijn Kant, Abraham J Valkenburg, Janesh Pillay, David M P van Meenen, Frederique Paulus, Marcus J Schultz, Virgil A S H Dalm, Eric C M van Gorp, Janke Schinkel, Henrik Endeman, PRoVENT- and PRoAcT-COVID Collaborative Group

**Affiliations:** 1 Department of Intensive Care Erasmus University Medical Center Rotterdam Netherlands; 2 Department of Viroscience Erasmus University Medical Center Rotterdam Netherlands; 3 Department of Infectious Diseases Public Health Service of Amsterdam Amsterdam Netherlands; 4 HIV Monitoring Foundation Amsterdam Netherlands; 5 Infectious Diseases Amsterdam University Medical Centers, location University of Amsterdam Amsterdam Netherlands; 6 Department of Intensive Care Amsterdam University Medical Centers, location Academic Medical Centre, University of Amsterdam Amsterdam Netherlands; 7 Laboratory of Experimental Intensive Care and Anesthesiology Amsterdam University Medical Centers, location Academic Medical Centre, University of Amsterdam Amsterdam Netherlands; 8 Department of Medical Microbiology & Infection Prevention Amsterdam University Medical Centers, location University of Amsterdam Amsterdam Netherlands; 9 Department of Internal Medicine and Intensive Care St Antonius Hospital Nieuwegein Netherlands; 10 Department of Intensive Care Maasstad Ziekenhuis Rotterdam Netherlands; 11 Department of Intensive Care Onze Lieve Vrouwe Gasthuis Hospital Amsterdam Netherlands; 12 Laboratory of Translational Intensive Care Erasmus University Medical Center Rotterdam Netherlands; 13 Department of Pulmonology Amphia Hospital Breda Netherlands; 14 Department of Intensive Care Amphia Hospital Breda Netherlands; 15 Department of Anesthesiology and Intensive Care Isala Clinics Zwolle Netherlands; 16 Department of Intensive Care University Medical Center Groningen, University of Groningen Groningen Netherlands; 17 Department of Pathology and Medical Biology Groningen Research Institute for Asthma and Chronic Obstructive Pulmonary Disease, University Medical Center Groningen University of Groningen Groningen Netherlands; 18 Department of Anesthesiology Amsterdam University Medical Centers, location Academic Medical Centre, University of Amsterdam Amsterdam Netherlands; 19 Center of Expertise Urban Vitality Faculty of Health Amsterdam University of Applied Sciences Amsterdam Netherlands; 20 Department of Immunology Erasmus University Medical Center Rotterdam Netherlands; 21 Division of Allergy & Clinical Immunology, Department of Internal Medicine Erasmus University Medical Center Rotterdam Netherlands; 22 Department of Internal Medicine, Erasmus University Medical Center Erasmus Netherlands; 23 See Acknowledgements

**Keywords:** COVID-19, corticosteroid, infectious diseases, virology

## Abstract

**Background:**

In hospitalized patients with COVID-19, the dosing and timing of corticosteroids vary widely. Low-dose dexamethasone therapy reduces mortality in patients requiring respiratory support, but it remains unclear how to treat patients when this therapy fails. In critically ill patients, high-dose corticosteroids are often administered as salvage late in the disease course, whereas earlier administration may be more beneficial in preventing disease progression. Previous research has revealed that increased levels of various biomarkers are associated with mortality, and whole blood transcriptome sequencing has the ability to identify host factors predisposing to critical illness in patients with COVID-19.

**Objective:**

Our goal is to determine the most optimal dosing and timing of corticosteroid therapy and to provide a basis for personalized corticosteroid treatment regimens to reduce morbidity and mortality in hospitalized patients with COVID-19.

**Methods:**

This is a retrospective, observational, multicenter study that includes adult patients who were hospitalized due to COVID-19 in the Netherlands. We will use the differences in therapeutic strategies between hospitals (per protocol high-dose corticosteroids or not) over time to determine whether high-dose corticosteroids have an effect on the following outcome measures: mechanical ventilation or high-flow nasal cannula therapy, in-hospital mortality, and 28-day survival. We will also explore biomarker profiles in serum and bronchoalveolar lavage fluid and use whole blood transcriptome analysis to determine factors that influence the relationship between high-dose corticosteroids and outcome. Existing databases that contain routinely collected electronic data during ward and intensive care admissions, as well as existing biobanks, will be used. We will apply longitudinal modeling appropriate for each data structure to answer the research questions at hand.

**Results:**

As of April 2023, data have been collected for a total of 1500 patients, with data collection anticipated to be completed by December 2023. We expect the first results to be available in early 2024.

**Conclusions:**

This study protocol presents a strategy to investigate the effect of high-dose corticosteroids throughout the entire clinical course of hospitalized patients with COVID-19, from hospital admission to the ward or intensive care unit until hospital discharge. Moreover, our exploration of biomarker and gene expression profiles for targeted corticosteroid therapy represents a first step towards personalized COVID-19 corticosteroid treatment.

**Trial Registration:**

ClinicalTrials.gov NCT05403359; https://clinicaltrials.gov/ct2/show/NCT05403359

**International Registered Report Identifier (IRRID):**

DERR1-10.2196/48183

## Introduction

### Background

The emergence of SARS-CoV-2 in Wuhan caused a global COVID-19 pandemic, with a high mortality and morbidity rate worldwide [1]. Over the past years, many efforts have been made to unravel the underlying complex pathophysiological mechanisms and explore various treatment options. Substantial variation exists in systemic and alveolar levels of inflammation between patients, contributing to the heterogeneous clinical course of the disease. This ranges from asymptomatic infection to life-threatening hypoxemic pneumonia. Corticosteroid therapy has the potential to inhibit disease progression and decrease the severity of clinical symptoms and is first-line immunosuppressive therapy in patients with COVID-19 with hypoxia [2].

The main reason for hospitalization of patients with COVID-19 is the requirement of supplemental oxygen therapy because of hypoxemia. In some patients, ground glass opacification and the beginning of consolidations on pulmonary CT scan are observed [3], accompanied by increased parameters of inflammation and coagulation in bronchoalveolar lavage fluid (BALF) and the systemic compartment [4,5]. In case of progressive respiratory failure, patients with COVID-19 will be admitted to the intensive care unit (ICU). These patients have a significantly decreased partial pressure of oxygen in arterial blood–fraction of inspiratory oxygen concentration ratio and frequently fulfill the Berlin criteria of moderate and severe acute respiratory distress syndrome (ARDS) [6]. Analysis of their inflammatory profiles shows a further elevation of proinflammatory cytokines, which is associated with increased mortality [7,8].

For decades, corticosteroids are given to treat patients with non–COVID-19 ARDS because of their strong anti-inflammatory effects [9]. Among patients with COVID-19, the Randomized Evaluation of Covid-19 Therapy (ie, RECOVERY) study showed that low-dose dexamethasone reduces mortality in patients requiring supplemental oxygen therapy or invasive mechanical ventilation but not in patients without respiratory support [10]. Current COVID-19 guidelines recommend a dose of 6 mg of dexamethasone per day for 10 days when oxygen therapy is required, regardless of the level of inflammation or severity of the disease [2]. Unfortunately, a proportion of patients still show progressive deterioration despite this treatment.

In these dexamethasone-unresponsive patients with COVID-19, corticosteroid therapy is sometimes escalated to a much higher dose. However, large heterogeneity in the type, timing, and dosing of escalated corticosteroid therapy exists [11]. High-dose corticosteroids are mainly administered during the late phase of the clinical course and in patients with the most severe disease, while earlier administration could be beneficial in preventing disease progression. However, the results of the studies on high-dose corticosteroid treatment for critically ill patients with COVID-19 are contradicting [12-15]. A recent meta-analysis compared high-dose corticosteroids versus low-dose corticosteroids in hospitalized patients with COVID-19 and reported no difference in mortality. Given the low level of evidence, inconclusive trial sequential analysis, and substantial heterogeneity, the authors stress the importance of future research to improve the certainty of evidence [16].

Treatment with high-dose corticosteroids may come at the cost of complications, such as secondary infections due to the immunosuppressive effects. To better predict the response to high-dose corticosteroids prior to therapy, additional diagnostic tools such as serum and BALF biomarker determination and whole blood transcriptome analysis could be helpful [17]. Implementation of these tools may result in a more precise and individualized approach to corticosteroid dosing in clinical practice.

Overall, the optimal dosing and timing of high-dose corticosteroids in hospitalized patients with COVID-19 remains uncertain. Here, we present the study design and methodological considerations of the SELECT (Steroids in Hospitalized Patients with COVID-19 in The Netherlands) study, a multicenter study that aims to study the most optimal dosing and timing of corticosteroid therapy to reduce the morbidity and mortality of hospitalized patients with COVID-19. In addition, we aim to provide a basis for personalized corticosteroid treatment regimens by exploring the predictive value of biomarkers and whole blood transcriptome analysis. We hypothesize that the effect of corticosteroid therapy on outcome is determined by the dosage and timing of administration, as well as the patient’s underlying inflammatory state.

### Study Aims

#### Work Package 1: High-Dose Corticosteroid Treatment in the Ward

**Figure 1:**
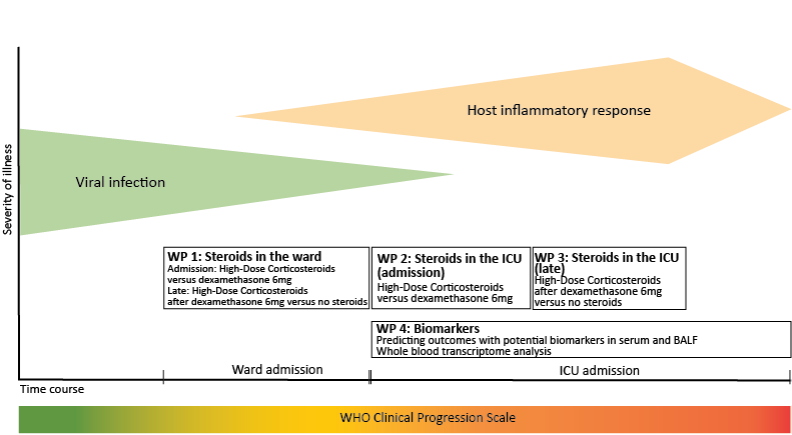
Integration of work packages in the Steroids in Hospitalized Patients with COVID-19 in The Netherlands (SELECT) study. On the y-axis, the viral and host inflammatory response are depicted. On the x-axis, the admission to the ward or intensive care unit (ICU) is shown, and below that, the World Health Organization (WHO) clinical progression scale is displayed as a measure of supplemental oxygen therapy. steroids: corticosteroids; HDS: high-dose corticosteroids; WP: work package.

Work package 1A (WP1A) is used to investigate whether initial treatment with high-dose corticosteroids compared to treatment with 6 mg dexamethasone per day for 10 days, or an equivalent corticosteroid, improves patient outcomes in the ward (Figure 1).

WP1B is used to investigate whether treatment with high-dose corticosteroids compared to no corticosteroids, after treatment with 6 mg dexamethasone per day for 10 days, improves patient outcomes in the ward.

#### Work Package 2: High-Dose Corticosteroid Treatment in the ICU

Work package 2 (WP2) is used to investigate whether initial treatment with high-dose corticosteroids compared to treatment with 6 mg dexamethasone per day for 10 days, or an equivalent corticosteroid, improves patient outcomes in the ICU.

#### Work Package 3: High-Dose Corticosteroid Treatment After Standard Corticosteroid Treatment in the ICU

Work package 3 (WP3) is used to investigate whether treatment with high-dose corticosteroids compared to no corticosteroids, after treatment with 6 mg dexamethasone per day for 10 days, improves patient outcomes in the ICU.

#### Work Package 4: Biomarker Profiles and Whole Blood Genome Transcriptomes in High-Dose Corticosteroid Treatment

Work package 4a (WP4a) is used to investigate whether biomarker profiles in serum and BALF act as response predictors for high-dose corticosteroids on the outcome.

WP4b is used to investigate whether whole blood transcriptome analysis acts as a response predictor for high-dose corticosteroids on the outcome.

## Methods

### Definition High-Dose Corticosteroids

High-dose corticosteroids are defined as every treatment with dexamethasone >6 mg daily or an equivalent corticosteroid (Table 1). Regarding prior and continuing use of corticosteroids for other conditions, corticosteroids are considered as part of the COVID-19–related intervention when the high-dose threshold is passed.

**Table 1:** Equivalent corticosteroids and dosages (total daily dosing)^a^.

Drug	Equivalent dose (mg)	Low-dose (mg)	High-dose (mg)
Dexamethasone	0.75	≤6	>6
Cortisone	25	≤200	>200
Hydrocortisone	20	≤160	>160
Prednis(ol)one	5	≤40	>40
Methylprednisolone	4	≤32	>32
Bethamethasone	0.75	≤6	>6

^a^This table shows the equivalent corticosteroids and dosages. The equivalent dose is derived from Farmacotherapeutisch Kompas [18].

### Study Design and Setting

**Figure 2:**
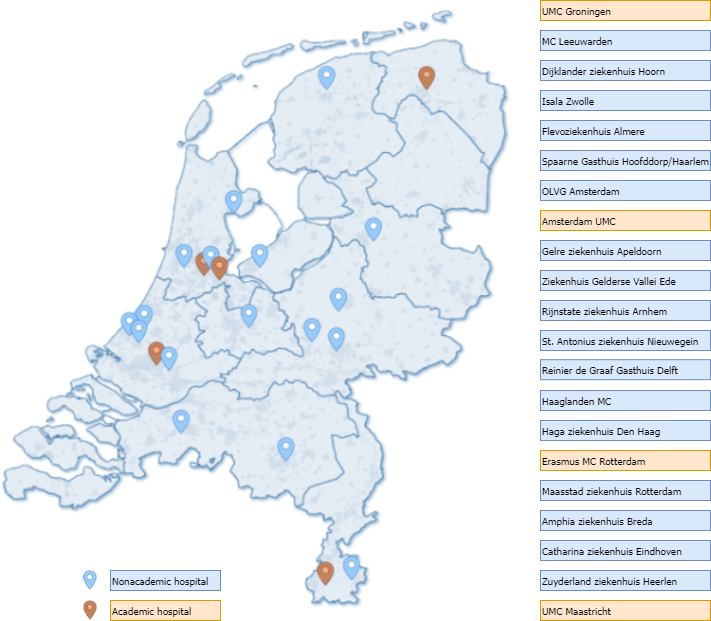
Participating hospitals of the Steroids in Hospitalized Patients with COVID-19 in The Netherlands (SELECT) study. UMC: University Medical Center. MC: Medical Center; OLVG: Onze Lieve Vrouwe Gasthuis.

The SELECT study is a retrospective cohort study with data from 22 hospitals in the Netherlands, including 4 academic and 18 nonacademic hospitals (Figure 2). Other hospitals may be included as the study progresses and we will use routinely collected electronic data from the participating hospitals. The data sets will contain data on hospitalized patients with COVID-19 from the date the first patient was admitted to the hospital in the Netherlands (March 1, 2020) until data transfer (expected May-December 2023). We will use the heterogeneity in therapeutic regimens in time (ie, first, second, and third epidemiological wave), centers (per protocol high-dose corticosteroids or not), and individual patients in the initial databases to address our aims. In a subpopulation of patients, we will use biobank material collected within the Collaboration on Identification, Understanding and Improved Management of patients with infectious diseases study of the Erasmus MC or collected at the Amsterdam University Medical Center (UMC) within the MERMAIDS-ARI, VIS cohort study or the Amsterdam UMC COVID-19 biobank.

### Patient Selection and Inclusion Criteria

**Figure 3:**
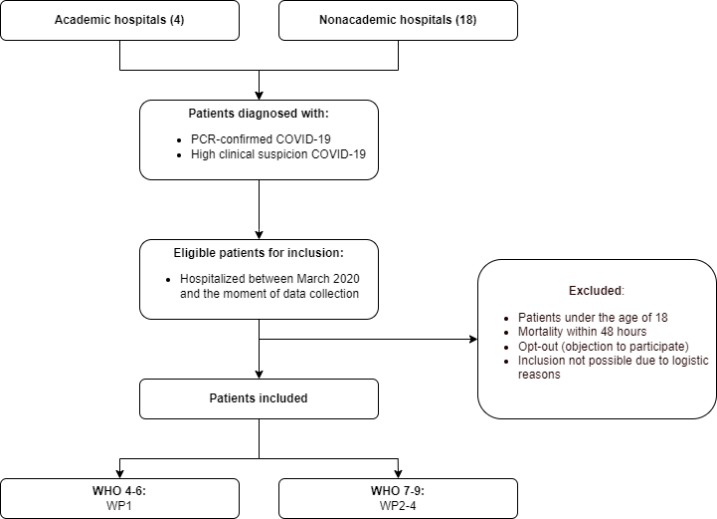
Patient inclusion. World Health Organization (WHO) classification: 0=uninfected, 1=asymptomatic; viral RNA detected, 2=symptomatic; independent, 3=symptomatic; assistance needed, 4=hospitalized; oxygen by mask or nasal prongs, 5=hospitalized; oxygen by mask or nasal prongs, 6=hospitalized; oxygen by noninvasive ventilation or high flow, 7=intubation and mechanical ventilation partial pressure of oxygen in arterial blood–fraction of inspiratory oxygen concentration (PaO2/FiO2) ratio of ≥150, 8=mechanical ventilation PaO2/FiO2 ratio of <150 or vasopressors, 9=mechanical ventilation PaO2/FiO2 ratio of <150 and vasopressors, dialysis or extracorporeal membrane oxygenation, 10=dead. PCR: polymerase chain reaction; WP: work package.

Adult patients (≥18 years) hospitalized with polymerase chain reaction–confirmed COVID-19 or high clinical suspicion of COVID-19 will be included (Figure 3). Pregnant patients and patients receiving corticosteroids for other conditions during or prior to hospital admission are eligible, although a prespecified sensitivity analysis excluding these patients will be performed. Patients who die within 48 hours after admission or patients who object to participate will be excluded from the study. In WP1 and WP4, we will include patients from all SARS-CoV-2 epidemic waves, and in WP2-3, we will include patients from the first and second waves only. Specific inclusion and exclusion criteria for each work package will be applied. In WP1A, patients will be included who are admitted to the ward with a World Health Organization clinical progression scale class of 4-5 [19] (ie, no oxygen or oxygen therapy via a cannula or [non-rebreather] mask). WP1B includes patients who have progressed to the World Health Organization clinical progression scale class of 5-6 (ie, noninvasive oxygen therapy, including conventional oxygen via cannula [non-rebreather] mask, high-flow nasal cannula, noninvasive continuous positive airway pressure, and noninvasive bilevel positive airway pressure) after in-hospital treatment with 10 days of 6 mg per day dexamethasone. The study population for WP2-4 consists of patients admitted to the ICU for a period exceeding 48 hours, who received invasive mechanical ventilation via an endotracheal tube, a tracheostomy, or extracorporeal membrane oxygenation during their ICU stay.

### Ethics Approval and Consent to Participate

The study will be conducted according to the principles of the Declaration of Helsinki (64th World Medical Association General Assembly, Fortaleza, Brazil, October 2013) and in accordance with Erasmus MC Research code, Netherlands Code of Conduct for Research Integrity, General Data Protection Regulation, Code of Conduct for Health Research (not subjected to the Medical Research Involving Human Subjects Act [non-WMO]), Code of Conduct for Responsible Use of Human Tissue (Non-WMO), and Medical Treatment Contracts Act. The study was approved by the Medical Research Ethics Committee from Erasmus University Medical Center (MEC-2022-0297) and the local review boards of all other participating centers. Regarding informed consent procedure: within the Erasmus MC, a consent waiver was provided by the Medical Research Ethics Committee for research including patients with COVID-19 due to the pandemic (exception due to emergency situation). Therefore, an opt-out informed consent procedure will be used in the participating centers, and subjects with registered objections to participate will be excluded from the study. This opt-out registration method was approved by the Medical Research Ethics Committee from Erasmus University Medical Center (MEC-2022-0297). Each center is responsible for appropriate informed consent procedures, which are covered in the data transfer agreements. Regarding the biobank data, only biobank material collected within the Collaboration on Identification, Understanding and Improved Management of patients with infectious diseases study of the Erasmus MC (METC number primary application: 2017-0417 and amendment 2020-0222) and samples collected at the Amsterdam UMC within the MERMAIDS-ARI (NL54834.018.15), VIS cohort study (NL73759.018.20), and the Amsterdam UMC COVID-19 biobank will be used.

### Data Collection

In WP1, variation in corticosteroid treatment protocols on the ward between centers is evaluated via questionnaires and extraction of data from the electronic patient record. The focus lies on collecting data from centers that use high-dose corticosteroids on the ward directly after admission and subsequently collecting data from centers with low-dose regimens as a comparison. For the data collection of ICU patients (WP2-3), a collaboration with 2 large COVID-19 study groups is started: the Practice of Ventilation in COVID-19 study (ie, PROVENT-COVID) study and the “Practice of Adjunctive Treatments in Intensive Care Unit Patients with Coronavirus Disease 2019” (PROACT-COVID) study [20,21]. The data of interest will be collected in Castor EDC [22], and it will be imported from each center via a secured data transfer system. Corticosteroid data will be collected on a daily basis including type, dose, and administration route. Regarding biobank WP4, a number of serum biomarker levels were already determined during standard COVID-19 care. In case of missing measurements, the stored samples (Tempus tubes) in the biobank can be used. The BALF was collected during routine COVID-19 care in the Amsterdam University Medical Centers and saved in aliquots at −80 °C for further analysis. SARS-CoV-2 viral load will be measured in BALF by using an in-house reverse transcription polymerase chain reaction. Subsequently, other commercially and generally available biomarkers might be determined in serum and BALF later by using a multiplex assay, such as Luminex. For the whole genome transcriptomes analysis, material was collected and saved in blood RNA Tempus tubes.

### Interventions

Patients were administered with >6 mg dexamethasone or an equivalent corticosteroid for a minimum of 3 consecutive days (Table 1). Our study permits the use of other general concurrent interventions, such as venous thromboembolism prophylaxis, fever and glucose control, fluid management, antibiotics, and antiviral drugs. The use of other immunomodulatory agents such as baricitinib, JAK inhibitors, and interleukin 6 pathway inhibitors, as well as antiviral drugs such as remdesivir, is allowed as part of a clinical trial or hospital policy. However, a sensitivity analysis will be performed excluding these patients.

### Outcomes

#### Primary Outcomes

Several outcome parameters will be studied for the different work packages based on the available data. In WP1A, the primary outcome of interest is hospital mortality in patients who do not receive high-flow nasal canule or invasive mechanical ventilation due to restrictions in care, either on medical grounds or advance directives of the patient. In patients who do not have restrictions in care, the need for high-flow nasal canule or invasive mechanical ventilation will be the primary outcome of interest (WHO severity 6-9). In WP1B, WP2, and WP3, 28-day survival and 28-day need for invasive mechanical ventilation will be studied as primary outcomes. In WP4, we will study the response to corticosteroids using the same outcomes.

#### Secondary Outcomes

Secondary outcomes for the ICU work packages include ICU mortality; in-hospital mortality; hospital length of stay (the number of days from the date of ICU admission to date of ICU discharge or death); mechanical ventilation duration (total duration of mechanical ventilation in days); and ventilator-free days, and alive at day 28. In all 4 work packages, we will evaluate the incidence of general systemic complications during the hospital stay as a secondary outcome, including myocardial infarction, deep venous thrombosis, pulmonary embolus, hyperglycemia, acute kidney injury, and sepsis. The definition of the various general systemic complications is shown in Table S1 in Multimedia Appendix 1.

### Statistical Analysis

Data will be analyzed on a convenience sample. No sample size calculations are considered for the study nor will they be provided post hoc [23]. The use of high-dose corticosteroids will be defined as a dichotomous variable (yes or no) in primary analysis and as a continuous variable in secondary analysis.

To study between-center variation in corticosteroid dosing strategy, the proportion of individuals who received high-dose corticosteroids will be compared across hospitals. Determinants of receiving high-dose corticosteroids will be evaluated using a multivariable logistic regression model with study center forced in the model. Whether the effects of significant determinants are different across centers will be tested through a determinant × center interaction term added for each determinant, separately.

In WP1, WP2, and WP3, several statistical analysis approaches will be considered to determine the association between high-dose corticosteroids and outcome, and statistical analyses will depend on the structure of the available data. A multivariable logistic regression model will be used with high-dose corticosteroids as exposure and survival at day 28 as an outcome. Covariates used for adjustment will include age, gender, BMI, smoking status, prior administration of corticosteroids during admission, comorbidity, sequential organ failure assessment score, oxygen saturation, partial pressure of oxygen in arterial blood–fraction of inspiratory oxygen concentration ratio, C-reactive protein, procalcitonin, ferritin, interleukin 6, D-dimer, lactate dehydrogenase, platelet count, lymphopenia, and level of respiratory support. Covariates can be added or removed based on insights from the data set or from the literature. A model might be constructed in which patients are matched on a propensity score based on their probability to receive high-dose corticosteroid treatment. Propensity scores will be determined using a logistic regression model with high-dose corticosteroids as a dependent variable and determinants of high-dose corticosteroids as independent variables. After matching, survival and mortality at day 28 and other outcome parameters will be compared between matched groups using logistic regression. A Cox proportional hazards model might be performed to determine the effect of high-dose corticosteroids on time to death while taking into account the days of administration using time-dependent covariates. Other approaches involving censoring weights for differential loss to follow-up (eg, marginal structural models) or competing risks (eg, joint models) might be explored.

Furthermore, we will use clinical and biological data to perform latent class analysis to search for subphenotypes, which respond more favorably to corticosteroid treatment. This heterogeneity of treatment effect will be evaluated by including the latent class analysis-derived subphenotype, corticosteroid exposure, and an interaction term of the 2 as c-variate in the above-described survival models. Heterogeneity of treatment effect is considered to be present if the interaction term has a *P* value of <.05.

Regarding WP4, patients are randomly selected and cases will be patients treated with high-dose corticosteroids, whereas controls will be patients who were not treated with high-dose corticosteroids. In WP4a, inverse probability treatment weighting and inverse probability of censoring weights will be used together in a marginal structural model. After we have analyzed the effect of high-dose corticosteroids on mortality and the other outcome measures, we will use this model to determine if the listed biomarkers modify the effect of high-dose corticosteroids on mortality.

In WP4b, we will identify transcriptome features that are differentially altered between cases and controls. Mean transcriptome levels will be compared using an unpaired *t*-test and *P* values will be adjusted for false-discovery rate using the Benjamini-Hochberg procedure. The log_2_ mean fold change of levels between cases and controls will also be calculated for each transcriptome. We will define transcriptome features as those with an adjusted *P* value of <.05 and a >2.0 or <–2.0 log_2_ mean fold change in level.

## Results

The study was funded in December 2021. As of April 2023, data have been collected for a total of 1500 patients, with data collection anticipated to be completed by December 2023. We expect the first results to be available in early 2024.

## Discussion

The SELECT study is a retrospective multicenter observational cohort study that aims to determine the most optimal dosing and timing of high-dose corticosteroid therapy in hospitalized patients with COVID-19 and to provide a basis for personalized treatment regimens using biomarkers and whole transcriptome analyses. Increasing insight in the pathophysiological pathways that underlie SARS-CoV-2 infection and the efficacy of high-dose corticosteroid therapy could lead to more precise targeting of high-dose corticosteroid therapy, and as a result, could improve patient outcomes.

The necessity of exploring this matter is underlined by the fact that there is currently no consensus regarding the administration of high-dose corticosteroid therapy in patients with COVID-19.

This study design has several strengths. First, no studies have been performed that evaluate the effect of high-dose corticosteroids throughout the entire clinical course of hospitalized patients with COVID-19. Our study will evaluate the effect of high-dose corticosteroids from hospital admission to the ward or ICU, until hospital discharge. Furthermore, our exploration of biomarker and gene expression profiles for targeted corticosteroid therapy represents a first step toward personalized corticosteroid therapy in COVID-19. By using existing research databases and biobanks, successful data collection is assured, and we will be able to include a large number of patients, which increases the generalizability of the results.

Due to the retrospective study design, some methodological considerations require further discussion. A major issue for any observational study is the fact that treatment modalities are not randomly assigned, but rather likely given based on specific patient characteristics or within-center protocols. This nonrandomized assignment is prone to bias by indication. To correct this bias, our initial analysis step will be the identification of factors that contribute to the allocation of corticosteroid treatment. Subsequently, we will examine several statistical approaches, such as the use of propensity scores at the patient level to minimize treatment indication bias.

The findings of this study could support COVID-19 guidelines and hospital protocols regarding the management of hospitalized patients with COVID-19. Thus far, the translation of predictive biomarkers and whole transcriptome analysis into an applicable bedside tool is highly challenging. We expect to provide valuable tools to guide the dosing and timing of high-dose corticosteroid therapy. Furthermore, the answers to our research questions might be extrapolated to patients with non–COVID-19 ARDS, as the use of high-dose corticosteroids is still a matter of debate in this group of patients. The mortality rate of non–COVID-19 ARDS is even higher than that of COVID-19 ARDS [24], emphasizing the need for improved treatment strategies. Nonetheless, more efforts are required to determine similarities and differences between patients with COVID-19 ARDS and those with non–COVID-19 ARDS to determine whether extrapolation from one group to the other is possible.

In conclusion, our study protocol presents a strategy to gain a better understanding of the optimal dosing and timing of high-dose corticosteroid therapy in hospitalized patients with COVID-19 and thereby improve patient outcomes.

## Appendix

Multimedia Appendix 1 Definition of general systemic complications.

Multimedia Appendix 2 Peer-review report by the COVID-19 Program - The Netherlands Organisation for Health Research and Development / ZorgOnderzoek Nederland (ZON) and the area Medical Sciences (MW) (ZonMw, Netherlands).

## Abbreviations

ARDS: acute respiratory distress syndrome
BALF: bronchoalveolar lavage fluid
ICU: intensive care unit
SELECT: Steroids in Hospitalized Patients with COVID-19 in The Netherlands
UMC: University Medical Center
WP: work package

## Data Availability

The data sets used and analyzed during the current study are available from the corresponding author upon reasonable request.
