# Supplementary material for: Optimal Dosing and Timing of High-Dose Corticosteroid Therapy in Hospitalized Patients With COVID-19: Study Protocol for a Retrospective Observational Multicenter Study (SELECT)
Source: JMIR Res Protoc. 2023 Jun 2;12:e48183. doi: 10.2196/48183 (PMC10276318; doi:10.2196/48183)
Supplement: Multimedia Appendix 2 [file resprot_v12i1e48183_app2.pdf]

Dear sir/madam,

These are the peer review reports from the grant application of the SELECT-study at ZonMW.

Kind regards,

Katrijn Daenen

ZonMw

ZonMw stimuleert gezondheidsonderzoek en zorginnovatie

Laan van Nieuw Oost-Indië 334
2593 CE Den Haag
Postbus 93245
2509 AE Den Haag
Telefoon 070 349 51 11
Fax 070 349 51 00
www.zonmw.nl
info@zonmw.nl

Erasmus MC
Intensive Care Volwassenen
De heer D. Gommers
Postbus 2040
3000 CA ROTTERDAM

Dossiernummer
50-56300-98-2115
Ons kenmerk
2021/37891/ZONMW

Datum
14 december 2021

Contactpersoon
Veerle Bos
Telefoon 070 515 03 13
e-mail covid19@zonmw.nl

Onderwerp
Subsidiebesluit over dossiernummer 10430102110010

Geachte heer Gommers,

Op 15 oktober 2021 heeft ZonMw uw subsidieaanvraag: '*Optimal dosing and timing of steroids in hospitalized patients with COVID-19'* ontvangen.
De commissie heeft tijdens de beoordelingsvergadering op 16 november 2021 uw aanvraag beoordeeld. De commissie heeft voorwaarden gesteld waarop u heeft gereageerd op 30 november middels een aanvullend wederhoor. Uw reactie is vervolgens positief beoordeeld door de commissie. In deze brief vindt u ons besluit en wat u van ons kunt verwachten. Daarnaast leest u wat wij van u verwachten.

Besluit
ZonMw besluit tot honorering van uw subsidieaanvraag. In overeenstemming met de door u ingediende begroting, ontvangt u een subsidie van maximaal € 533.180,- voor de duur van maximaal 24 maanden. Dit bedrag is inclusief eventuele btw.
ZonMw verleent deze subsidie onder de voorwaarde dat **binnen 6 maanden** na de verzenddatum van deze brief een finale conceptversie (goedgekeurd door partijen maar nog niet ondertekend) van de consortiumovereenkomst(en) per email door ZonMw is ontvangen. Indien ZonMw de betreffende overeenkomst(en) niet accepteert, kan ZonMw besluiten dat er geen subsidie wordt verleend.

Onderbouwing besluit
De commissie realiseert zich dat deze aanvraag is ingediend binnen een hele bijzondere dynamiek en in een hele korte tijdspanne. Zij wil bij deze haar grote waardering uitspreken naar de projectgroep voor het realiseren van een aanvraag met betrokken partners binnen dit tijdsbestek.

De beoordelingscommissie van het COVID-19 deelprogramma behandeling heeft positief geadviseerd over uw aanvraag. Dit oordeel is gebaseerd op uw aanvraag, de beoordeling door de commissie, het wederhoor, het interview tijdens de commissievergadering en het aanvullend wederhoor. Het advies van de commissie is naar het oordeel van ZonMw op zorgvuldige wijze tot stand gekomen. ZonMw heeft dit advies overgenomen en ten grondslag gelegd aan haar beslissing.

Alle subsidieaanvragen zijn beoordeeld op kwaliteit en relevantie voor het programma en de subsidieoproep: COVID-19 behandeling kennishiaten (FMS kennisagenda COVID-19). Uw aanvraag richt zich op het kennishiaat: *Wat is de meest optimale vorm, timing en dosis van steroïd behandeling gericht op verbeteren van morbiditeit en mortaliteit van in het ziekenhuis opgenomen COVID-19 patiënten?*

Oordeel over relevantie
De commissie heeft het volgende oordeel over de relevantie van uw subsidieaanvraag: **zeer relevant**

Het relevantieoordeel is op de volgende argumenten gebaseerd:
- Corticosteroïden zijn een zeer succesvolle medicinale behandeling tegen COVID-19 bij patiënten die zijn opgenomen in het ziekenhuis. Er zijn echter nog onduidelijkheden over de dosering en timing van deze behandeling. Het onderzoek zal met behulp van retrospectieve data die verkregen zijn uit de eerdere COVID-19 golven trachten een onderbouwing te geven voor het meest optimale behandelprotocol. Daarmee draagt dit onderzoek bij aan de invulling van een belangrijk kennishiaat dat is geformuleerd door de FMS in de COVID-19 kennisagenda.
- De onderzoeksgroep heeft een unieke positie om de kennisvraag te beantwoorden en heeft een breed draagvlak om de resultaten in richtlijnen om te zetten.

Oordeel over kwaliteit
De commissie heeft het volgende oordeel over de kwaliteit van uw subsidieaanvraag: **goed**

Het kwaliteitsoordeel is op de volgende argumenten gebaseerd:
- De opzet en methodologie die wordt voorgesteld in het onderzoek is van goede kwaliteit. De commissie is daarom van mening dat de studie op korte termijn resultaten kan opleveren die van belang zijn voor de behandeling van patiënten met ernstige COVID-19.
- Het projectteam wordt door de commissie beoordeeld als zeer deskundig. De commissie waardeert de aanvulling van statistische expertise in de eerste 12 maanden.
- De adviespunten van de commissie zijn adequaat verwerkt in het aanvullend wederhoor.

Aanvullende voorwaarden/aanbevelingen voor de uitvoer van uw project
Graag zien we het volgende aspect terug in uw voortgangs- en eindverslagen:

- De gekozen studieopzet geeft voornamelijk inzicht in de dosering en minder in de optimale timing van een behandeling met corticosteroïden. Om deze reden verzoekt de commissie u om het timing aspect minder sterk te benadrukken.

Wij vragen u extra aandacht te hebben voor implementatie, opschaalbaarheid en borging van resultaten uit uw project. ZonMw wil de nieuwe kennis zo snel mogelijk beschikbaar maken voor het veld en de praktijk. U kunt, buiten reguliere rapportages om, door het programmasecretariaat gevraagd worden om informatie en updates over de voortgang van uw project (mondeling, schriftelijk of als een presentatie) aan te leveren. We gaan ervan uit dat u uw medewerking daaraan verleent.

Samenwerking/bijdragen van derden
Op de ZonMw webpagina Subsidies en Samenwerking/bijdragen van derden vindt u meer informatie, voorbeelden van en de voorwaarden voor het opstellen van een consortium- en/of sponsorovereenkomst. Wij verzoeken u deze voorbeeldovereenkomsten en informatie te gebruiken bij het opstellen van de betreffende overeenkomst, voor zover van toepassing. Maak hierbij duidelijk wat de wijzigingen ten opzichte van de voorbeeldovereenkomst zijn.
De overeenkomst wordt beoordeeld op conformiteit aan het toepasselijke Europese staatssteunrecht, de algemene subsidiebepalingen van ZonMw en de voorwaarden van de oproep. Acceptatie door ZonMw van de concept consortium- en/of sponsorovereenkomst ontslaat de subsidieontvanger niet van zijn eigen verantwoordelijkheid met betrekking tot het voorkomen van schending van het Europese staatssteunrecht. Na acceptatie van de concept consortium- en/of sponsorovereenkomst door ZonMw (voor zover die is opgevraagd) en voor de start van het project stuurt u de volledig ondertekende overeenkomst naar ZonMw. De startdatum van het project mag niet eerder zijn dan de datum bij de laatste handtekening op de overeenkomst.

Opdrachtverlening
Als er sprake is van inhuur van/opdrachtverlening aan derden dan dienen deze afspraken te voldoen aan de voorwaarden voor inhuur/opdrachtverlening zoals vermeld op de ZonMw webpagina Subsidies en Samenwerking/bijdragen van derden.

Open science - Datamanagement
Om data uit uw project ook in de toekomst herbruikbaar te laten zijn dient u een datamanagementplan op te stellen. Informatie over hoe u dat moet doen, vindt u op de ZonMw website via www.zonmw.nl/en/research-and-results/fair-data-and-data-management/data-management-in-your-project/. Volg vanaf deze link de stappen in de procedure.
Dien **uiterlijk 3 maanden** na verzenddatum van deze brief uw datamanagementplan en (voorlopige) kerngegevens in. Beide kunt u sturen naar covid19@zonmw.nl. Indien u geen dataverzameling opbouwt, kunt u dat melden bij het programmateam.

Open science - Open Access
Alle publicaties die voortkomen uit wetenschappelijk onderzoek dat geheel of gedeeltelijk door ZonMw gesubsidieerd is, dienen direct (zonder embargo) Open Access beschikbaar gesteld te worden, overeenkomstig met het ZonMw Open Access beleid. ZonMw accepteert verschillende Open Access routes. Naast artikelen, moedigt ZonMw ook aan om andere type wetenschappelijke publicaties Open Access beschikbaar te stellen.
U dient te rapporteren over uw producten in ProjectNet en het voortgangs- en eindverslag. Voor meer informatie over het ZonMw Open Access beleid en het beleidsdocument zie: www.zonmw.nl/nl/over-zonmw/open-science-fair-data/open-access/.

Creative Commons licentie
Publicaties dienen gepubliceerd te worden onder toepassing van de *Creative Commons* Naamsvermelding (CC BY 4.0 licentie). Dit betekent dat op de auteursversie van deze publicaties (tenzij in de subsidieoproep anders is bepaald) een onherroepelijke, niet-exclusieve CC BY licentie van toepassing is. U dient van het bestaan van deze licentie melding te maken aan elke partij met wie u in overleg treedt omtrent publicatie van (een deel van de) projectresultaten. ZonMw is te allen tijde gerechtigd aan derden melding te maken van deze licentie. Indien de auteur het noodzakelijk acht om af te wijken van de CC BY 4.0 licentie, dient u de alternatieve keuze te onderbouwen en ter goedkeuring voor te leggen aan ZonMw.

Europe Pubmed Central (EPMC)
Voor alle subsidierondes gepubliceerd vanaf 01-01-2021 vereist ZonMw dat projectleiders tenminste een kopie (*Version of Record* of het *Author Accepted Manuscript*) van artikelen voortkomend uit hun ZonMw project deponeren in EPMC (zelfdeponering of via uitgever).
In verband met de koppeling die in EPMC wordt gemaakt tussen publicaties en subsidie en project informatie van door ZonMw gefinancierde projecten, worden met enige regelmaat algemene projectgegevens en enkele persoonsgegevens gelinkt aan het project (naam projectleider, e-mailadres (*enkel voor het maken van de koppeling, dit wordt niet verder gedeeld of gebruikt*), organisatie en afdeling) gedeeld met EMBL-EBI, de beheerder van EPMC. ZonMw heeft hiervoor een officiële *data sharing agreement* afgesloten. Mocht u hier bezwaar tegen hebben, dan vernemen wij dit graag *uiterlijk twee weken* na ontvangst van deze toekenningsbrief. Indien wij niets van u vernemen, gaan wij ervan uit dat u akkoord bent met deze verwerking van uw persoonsgegevens.

Open Access publicatiekosten
Indien u Open Access publicatiekosten heeft opgenomen in de projectbegroting dient u t.z.t. de naam van het journal en een bewijs van betaling Article Processing Charges te mailen naar covid19@zonmw.nl en openscience@zonmw.nl.’

Kennisbenutting
ZonMw wil weten wat er met de resultaten van uw project gebeurt. Geef in de voortgangsverslagen aan met wie u samenwerkt en welke verspreiding- en implementatieactiviteiten u onderneemt. Geef aan of de resultaten direct geïmplementeerd worden in de praktijk. Spelen de resultaten een rol bij het maken van beleid, onderwijs, een volgende stap in een wetenschappelijke carrière of vormen de resultaten een basis voor een nieuw project? U bent verplicht om ZonMw tot 4 jaar na afronding via ProjectNet te informeren over het gebruik van de resultaten en publicaties.

Verklaringen en vergunningen
ZonMw keert pas een voorschot uit als aan alle eisen voor het uitvoeren van het onderzoek is voldaan. Wij raden u aan eventuele procedures hiervoor tijdig te starten. Denk bijvoorbeeld aan een positief oordeel van een erkende medisch-ethische toetsingscommissie (METC), de Centrale Commissie Mensgebonden Onderzoek (CCMO), een projectvergunning van de Centrale Commissie Dierproeven (CCD), of een vergunning krachtens de Wet op het Bevolkingsonderzoek (WBO). U kunt bij de betreffende instanties nagaan of uw project dergelijke verklaringen of vergunningen nodig heeft.

Licentie of overdracht van projectresultaten
Bij de uitvoering van uw project en het gebruik van (toekomstige) resultaten dienen de principes van Maatschappelijk Verantwoord Licentiëren te worden toegepast.

Integriteit
Het project dient op een goede en integere manier uitgevoerd te worden. Hiervoor gelden onder andere de volgende principes: eerlijkheid, zorgvuldigheid, transparantie, onafhankelijkheid en verantwoordelijkheid. Deze principes zijn vastgelegd in de Nederlandse gedragscode wetenschappelijke integriteit. Indien bij een door ZonMw gefinancierd project een (mogelijke) integriteitsschending plaatsvindt, dient ZonMw hier zonder uitstel van op de hoogte te worden gesteld. Alle relevant documenten over de integriteitsschending dienen aan ZonMw te worden overhandigd.

Start project
Het project dient zo spoedig mogelijk te beginnen.

Toelichting op hoogte subsidiebedrag

- Conform uw ingediende begroting.
- Van het subsidiebedrag is € 5.000,- gereserveerd voor Open Access.
- Van de verschillende posten dient bij de eindafrekening een specificatie te worden aangeleverd.

Betaling van de subsidie
De subsidie zal via voorschotbedragen aan u worden overgemaakt. De betalingen zijn afhankelijk van de ontvangst en goedkeuring van voortgangsverslagen en de eindverantwoording van uw project. U ontvangt het eerste voorschot als wij het ingevulde meldingsformulier hebben ontvangen.

Eindverantwoording
Binnen 13 weken na afloop van uw project dient u een eindverantwoording in. Dit is een inhoudelijk eindverslag en de financiële eindverantwoording. De financiële verantwoording dient een specificatie van de verschillende posten te bevatten. Na ontvangst en goedkeuring van de eindverantwoording vindt de definitieve subsidievaststelling en afrekening plaats.

Tussentijdse wijzigingen
Laat het ons weten als er tussentijds iets in uw plan of uw organisatie verandert. Verandering in de opzet, planning, begroting of organisatie kan gevolgen hebben voor uw subsidie. Pas na goedkeuring door ZonMw zijn eventuele wijzigingen toegestaan.

Wat verwachten wij nu van u?
ZonMw kan u een voorschot voor de eerste periode van uw project verstrekken. Hiervoor dient u:
- **Binnen 4 weken** na de verzenddatum van deze brief bijgevoegd meldingsformulier ingevuld retour te sturen.
- **Zo spoedig mogelijk** na de verzenddatum van deze brief een Nederlandse publiekssamenvatting in ProjectNet in te vullen (maximaal 1000 tekens, inclusief spaties). ZonMw publiceert alle gehonoreerde projecten op haar website met een Nederlandse samenvatting in eenvoudige taal. Deze is bedoeld voor een breed geïnteresseerd publiek met verschillende achtergronden. Zie de schrijfwijzer op http://www.zonmw.nl/nl/over-zonmw/logo-huisstijl.
- U dient **6 maanden na de startdatum** van uw project een kopie van de door alle partijen ondertekende finale versie van de consortium- en/of sponsorovereenkomst aan ZonMw toe zenden.

Vragen
Heeft u nog vragen? Neemt u dan gerust contact op met de medewerker die in het briefhoofd vermeld staat. Dat kan via e-mail: covid-19@zonmw.nl of via telefoonnummer: 070 515 03 13. Houd uw projectnummer bij de hand zodat wij u snel kunnen helpen. Wij verzoeken u in het vervolg uw nieuwe projectnummer te gebruiken: **10430102110010**.

Bezwaar of klacht?
Bent u het niet eens met dit besluit? Dan heeft u de mogelijkheid om een bezwaarschrift in te dienen. Mocht u dat overwegen adviseren wij u eerst uw contactpersoon te benaderen. Een bezwaarschrift moet u binnen 6 weken na de verzenddatum van deze brief sturen naar het bestuur van ZonMw, t.a.v. Commissie Bezwaarschriften ZonMw, Postbus 93 245, 2509 AE Den Haag.

Als u ontevreden bent over de wijze waarop ZonMw uw aanvraag heeft behandeld, kunt u ons dit laten weten of een klacht indienen. Meer informatie vindt u op de ZonMw website via www.zonmw.nl/signalerenklagenbezwaarmaken.

Ik feliciteer u met de honorering van uw subsidieaanvraag en wens u succes bij de uitvoering van uw project!

Met vriendelijke groet,
namens het bestuur van ZonMw,

Véronique Timmerhuis
Algemeen directeur

Bijlagen
Subsidieverplichtingen
Meldingsformulier start project 10430102110010

Kopie
Mevrouw J. Schinkel, Amsterdam UMC - locatie AMC
De heer dr. H. Endeman, Erasmus MC

Subsidieprogramma / Subsidy programme	:	**COVID-19 Programma**
Dossiernummer / Dossier number	:	**50-56300-98-2115**
Aanvrager / applicant	:	**Dr. H. Endeman**
Projecttitel / Project title	:	**Optimal dosing and timing of steroids in hospitalized patients with COVID-19**
Beoordelingscode / Assessment code	:	**B.2021.013BC**

1. Kwaliteitscriteria

Doelstelling en vraag- of taakstelling

Denk daarbij aan:
- Helderheid, concreetheid vraag- of taakstelling en doelstelling. Is de doelstelling SMART (specifiek, meetbaar, acceptabel, realistisch en tijdsgebonden) geformuleerd?
- Is helder wat de beoogde opbrengsten zijn en voor wie deze zijn bedoeld?

Plan van aanpak

Denk daarbij aan:
- Theoretische of empirische onderbouwing
- Adequaatheid van plan van aanpak voor de vraagstelling/doelstelling
- Geschiktheid van gekozen methoden/analyses (design, studiepopulatie, data-bronnen)
- Is er een heldere powerberekening toegevoegd, indien van toepassing?

Haalbaarheid

Denk daarbij aan:
- Is het een realistische verwachting dat het project snel kan starten?
- Is het onderzoek haalbaar in de huidige pandemiesituatie?
- Is de recruteringsstrategie en inclusie verwachting realistisch?
- Is de beschikbaarheid van faciliteiten/mensen realistisch?
- Zijn de verwachte resultaten, fasering en tijdsplanning realistisch?

Kennisoverdracht en implementatie

Denk daarbij aan:
- goede mix van activiteiten voor kennisoverdracht en/of bestendiging;
- mate waarin de betrokkenheid van gebruikers en doelgroepen (inclusief patiëntenvertegenwoordigers) aandacht krijgt;
- is de implementatiestrategie duidelijk?

2. Samenvattend oordeel kwaliteit

Legenda: ZG (zeer goed), G (goed), V (voldoende), M (matig), O (onvoldoende)

2.1 Sterke punten kwaliteit: toelichting naar de aanvragers

Het voorstel bestaat waarschijnlijk uit meerdere werkpakketten. Maak in uw beoordeling per punt duidelijk op welk werkpakket uw commentaar betrekking heeft.

Benoem sterke punten per onderdeel:

1.1. Probleemstelling en doelstellingen

1.2. Plan van aanpak

1.3. Haalbaarheid

1.4. Kennisoverdracht en implementatie

Zeer relevant onderzoek over timing en dosering van hoog-gedoseerde steroïden in de behandeling van COVID-19 patiënten met complex beloop.
WP1: dexa unresponsive categorie. Belangrijke groep, gekozen behandeingsgroepen zijn logisch en niet nodeloos complex. Twee groepen zal de analyse helpen. Gekozen aantal patiënten is realistisch.
WP2 en 3: hoog gedoseeerd steroiden in plaats van dexa of na dexa. Gezien het geprotraheerde beloop van de ziekte bij sommige geinfecteerden is vooral WP3 zeer interessant. Hoog gedoseerde steroiden worden al gegeven door een aantal ICU's (misschien alle), maar de duidelijke vraagstelling en het plan van aanpak van deze studie zal helpen met de wetenschappelijke onderbouwing van het beleid. Deze studie zal helpen bij het identificeren van patiënten die hier baat van kunnen hebben en in welke fase van de ziekte deze medicatie verschil kan maken.
WP4: biobank voor het identificeren van markers die de outcome voorspellen is zeer waardevol.

2.2 Zwakke punten kwaliteit: toelichting naar de aanvragers

Het voorstel bestaat waarschijnlijk uit meerdere werkpakketten. Maak in uw beoordeling per punt duidelijk op welk werkpakket uw commentaar betrekking heeft.

Benoem zwakke punten per onderdeel:

1.1. Probleemstelling en doelstellingen

1.2. Plan van aanpak

1.3. Haalbaarheid

1.4. Kennisoverdracht en implementatie

Bij alle 4 WPs worden geen markers benoemd die al bestaan op het moment dat de patient wordt opgenomen, ofwel op de afdeling, ofwel op de IC. Het is mij daarom niet geheel duidelijk hoe de verschillende ziektefases waarin de patiënt zich mogelijk bevindt worden vastgesteld. Er wordt in de beschrijving van de studie geen diagnostiek voorgesteld die hierbij kan helpen, zoals het gebruik van voor de kliniek gebruikelijke chemische parameters zoals CRP of procalcitonine. Ook wordt geen beeldvorming meegenomen in de huidige beschrijving.
De WPs van de studies die worden voorgesteld gaan uit van de situatie: "patiënt krijgt al dexa en wat doen we als hij slechter wordt".
Op zich is dit een redelijke vraag, maar hierbij kunnen we niet meer kritisch zijn op de timing van de eerste dexa, terwijl dit heel erg relevant zal worden tijdens de volgende COVID-19 golf.
Met de vaccinatie graad die in Nederland bereikt is, zullen patiënten die de komende maanden worden opgenomen voor de vuist weg kunnen worden ingedeeld in: 1) redelijk gezonde vaccinatieweigeraars, en 2) mensen die door de vaccinatie onvoldoende beschermd waren tegen ernstige gevolgden van COVID-19.
Die laatste groep kan zich anders gedragen dan de eerste, met mogelijk een langere virale fase en een meer of minder uitgesproken begin van de inflammatoire fase omdat bijv. immuunsuppressie is/wordt gestopt.
Ik verwacht dat het relatieve aandeel van de immuun-gecompromiteerden of "immunologically challanged" patiënten groep groter zal zijn dan bij de eerdere golven.
Mijn advies aan de onderzoekers is om baseline markers, zoals SARS-CoV-2 Ct waardes bij opname en overplaatsting, infectie parameters, medicatie voorgeschiedenis, en duur van de ziekte mee te nemen in de studie om er voor te zorgen dat er analyseerbare groepen ontstaan. Het is onduidelijk of iemand bij wie vorige week de metotrexaat of MMF is gestopt nu meer baat heeft bij hoog gedoseerde steroïden. Het is onduidelijk of de hematologie patiënt met rituximab zich op dit moment van O2 behoefte wel bevindt in fase 2 of 3.
Op dit moment lijkt het erop dat deze baseline characteristics in WP4 worden meegenomen, maar daarmee wordt

niet geborgd dat voldoende deelnemers worden geincludeerd die zich mogelijk in een andere fase van de ziekte bevinden waar de steroïden behandeling meer of minder zinvol is.

2.3 Samenvattend oordeel kwaliteit

ZG	G	V	M	O
	X			

Geef een samenvattend oordeel over de kwaliteit van de aanvraag.

Zeer goed: Het voorstel adresseert de kwaliteitscriteria zeer goed, en ik heb eventueel een aantal suggesties voor kleine verbeteringen

Goed: Het voorstel adresseert de kwaliteitscriteria goed, maar een aantal verbeteringen zijn noodzakelijk

Voldoende: In het algemeen zijn de kwaliteitscriteria geadresseerd, maar er zijn significante tekortkomingen die gecorrigeerd dienen te worden

Matig: Het voorstel vertoont tekortkomingen met betrekking tot de kwaliteitscriteria, verbetering is noodzakelijk

Onvoldoende: Het voorstel vertoont ernstige tekortkomingen met betrekking tot de kwaliteitscriteria

3. Relevantiecriteria

Beantwoorden kennishiaat
Het onderzoek levert de urgent benodigde kennis om het in de kennisagenda COVID-19 beschreven kennishiaat te beantwoorden. Geef indien mogelijk aan welke werkpakketten het meest urgent en welke het minst urgent zijn voor het beantwoorden van de kennisvraag.

Impact.
Het onderzoek is van groot belang omdat het gedurende de pandemie, en de nasleep daarvan, impact kan hebben op de behandeling van COVID-19 patiënten; of bijdraagt aan het beter voorbereid zijn op een eventuele volgende pandemie.

Implementeerbare tussenresultaten.
De aanvraag moet overtuigend onderbouwen dat onderzoek op korte termijn leidt tot implementeerbare (tussen)resultaten (tenminste tijdens de pandemie en daarnaast ook voor de lange termijn).

Unieke
positie onderzoeksveld en toegevoegde waarde naast ander onderzoek, er wordt zoveel mogelijk gebruik gemaakt van lopend onderzoek en/of registraties en cohorten

Nieuw onderzoek
Het onderzoek wordt niet al (inter)nationaal uitgevoerd (aanvrager moet dit overtuigend onderbouwen), of de aanvrager moet overtuigend onderbouwen dat onderzoek in Nederland nodig is naast internationaal onderzoek.

Geschikte onderzoeksgroep
Er is een competente onderzoeksgroep/ consortium/ samenwerkingsverband beschikbaar om dit op korte termijn op te pakken. In het consortium is een goede balans tussen academische, STZ en perifere ziekenhuizen en waar van toepassing samenwerking met eerstelijnsorganisaties. Kennishiaat wordt zoveel mogelijk in samenhang opgepakt, door de zorgketen heen.

Voortbouwen op lopend onderzoek en bestaande cohorten en/of databestanden
In deze ronde is in principe alleen financiering beschikbaar voor projecten die voortbouwen op lopend onderzoek en/of gebruik maken, koppelen en doorontwikkelingen van bestaande cohorten en/of databestanden.

Draagvlak
Bij beroepsgroepen en zorginstellingen is voldoende geborgd

Patiëntenparticipatie
Patiëntenparticipatie in het consortium is vereist, als partner vanaf protocol ontwikkeling. Met participatie wordt concreet bedoeld het raadplegen, advies inwinnen, samenwerken en/of laten (mee)beslissen van betrokkenen.

Diversiteit
Aanvragers dienen in de opzet, uitvoering, analyse en rapportage van resultaten onderscheid te maken tussen

mannen en vrouwen en andere relevante subgroepen op basis van diversiteit. Indien diversiteit niet relevant is voor het voorgestelde onderzoek, wordt dit voldoende onderbouwd?

4. Samenvattend oordeel relevantie

Legenda: ZR (Zeer relevant), R (Relevant), LR (Laag relevant)

4.1 Sterke punten relevantie: toelichting naar de aanvragers

Het voorstel bestaat waarschijnlijk uit meerdere werkpakketten. Maak in uw beoordeling per punt duidelijk op welk werkpakket uw commentaar betrekking heeft

Benoem sterke punten per onderdeel en benoem duidelijk over werkpakket het gaat:

3.1. Beantwoorden kennishiaat

3.2. Impact

3.3. Implementeerbare tussenresultaten

3.4. Unieke positie

3.5. Nieuw onderzoek

3.6. Geschikte onderzoeksgroep

3.7. Voortbouwen op lopend onderzoek en bestaande cohorten en/of databestanden

3.8. Draagvlak

3.9. Patientenparticipatie

3.10. Diversiteit

Dit onderzoek is relevant, vooral voor het beantwoorden van vragen over de timing en het nut van hoog gedoseerde steroïden in de gemiddelde, immuuncompetente patiënt met COVID-19.
Dit onderzoek zal impact hebben en de resultaten zullen snel geïmplementeerd kunnen worden.
De vragen die gesteld worden en die beantwoord zullen worden zijn zeer actueel en zullen internationaal gezien worden. De onderzoeksgroep is zeker zeer geschikt om deze vragen te onderzoeken en de onderzoekers hebben breed draagvlak onder hun collega's om de resultaten in richtlijnen om te zetten.
De onderzoeksgroep is divers en er is vertegenwoordiging vanuit verschillende groepen.

4.2 Zwakke punten relevantie: toelichting naar de aanvragers

Het voorstel bestaat waarschijnlijk uit meerdere werkpakketten. Maak in uw beoordeling per punt duidelijk op welk werkpakket uw commentaar betrekking heeft

Benoem zwakke punten per onderdeel:

3.1. Beantwoorden kennishiaat

3.2. Impact

3.3. Implementeerbare tussenresultaten

3.4. Unieke positie

3.5. Nieuw onderzoek

3.6. Geschikte onderzoeksgroep

3.7. Voortbouwen op lopend onderzoek en bestaande cohorten en/of databestanden

3.8. Draagvlak

3.9. Patientenparticipatie

3.10. Diversiteit

Vooruitlopend op wie de mensen zullen zijn die in de komende maanden in het ziekenhuis zullen belanden met COVID-19, is het sterk geadviseerd om mensen met immunologische stoornissen of immuuncompromiteerdende medicatie bewust te includeren in aantallen die analyse van deze groepen mogelijk te maken.
De timing van dexa in relatie tot de verschillende fases van de ziekte is nog niet duidelijk, net zo min als de effecten van het stoppen van de immuunsuppressie bij het bijvoorbeeld niet klaren van een infectie.
In de toekomst zullen mensen die niet goed beschermd zijn tegen SARS-CoV-2 infecties) na vaccinatie en na een doorgemaakte infectie) een probleem blijven. Wat mij betreft zeer de moeite waard!

4.3 Samenvattend oordeel relevantie

ZR	R	LR
	X	

Geef een samenvattend relevantie oordeel

5. Begroting

Legenda: TH (Te hoog), R (reëel), TL (te laag)

5.1 Begroting

TH	R	TL
	X	

In hoeverre is het budget realistisch gezien de geplande activiteiten? Is de toelichting op de begroting voldoende helder?

Gezien het grote aantal deelnemers is dit een reëel budget. Er is relatief weinig begroot voor laboratorium analyses en veel begroot voor statische analyse.

Subsidieprogramma / Subsidy programme	:	**COVID-19 Programma**
Dossiernummer / Dossier number	:	**50-56300-98-2115**
Aanvrager / applicant	:	**Dr. H. Endeman**
Projecttitel / Project title	:	**Optimal dosing and timing of steroids in hospitalized patients with COVID-19**
Beoordelingscode / Assessment code	:	**B.2021.013BD**

1. Kwaliteitscriteria

Doelstelling en vraag- of taakstelling

Denk daarbij aan:
- Helderheid, concreetheid vraag- of taakstelling en doelstelling. Is de doelstelling SMART (specifiek, meetbaar, acceptabel, realistisch en tijdsgebonden) geformuleerd?
- Is helder wat de beoogde opbrengsten zijn en voor wie deze zijn bedoeld?

Plan van aanpak

Denk daarbij aan:
- Theoretische of empirische onderbouwing
- Adequaatheid van plan van aanpak voor de vraagstelling/doelstelling
- Geschiktheid van gekozen methoden/analyses (design, studiepopulatie, data-bronnen)
- Is er een heldere powerberekening toegevoegd, indien van toepassing?

Haalbaarheid

Denk daarbij aan:
- Is het een realistische verwachting dat het project snel kan starten?
- Is het onderzoek haalbaar in de huidige pandemiesituatie?
- Is de recruteringsstrategie en inclusie verwachting realistisch?
- Is de beschikbaarheid van faciliteiten/mensen realistisch?
- Zijn de verwachte resultaten, fasering en tijdsplanning realistisch?

Kennisoverdracht en implementatie

Denk daarbij aan:
- goede mix van activiteiten voor kennisoverdracht en/of bestendiging;
- mate waarin de betrokkenheid van gebruikers en doelgroepen (inclusief patiëntenvertegenwoordigers) aandacht krijgt;
- is de implementatiestrategie duidelijk?

2. Samenvattend oordeel kwaliteit

Legenda: ZG (zeer goed), G (goed), V (voldoende), M (matig), O (onvoldoende)

2.1 Sterke punten kwaliteit: toelichting naar de aanvragers

Het voorstel bestaat waarschijnlijk uit meerdere werkpakketten. Maak in uw beoordeling per punt duidelijk op welk werkpakket uw commentaar betrekking heeft.

Benoem sterke punten per onderdeel:

1.1. Probleemstelling en doelstellingen

1.2. Plan van aanpak

1.3. Haalbaarheid

1.4. Kennisoverdracht en implementatie

1.1 Probleemstelling en doelstelling:

WP 1-4:
Het betreft een relevante onderzoeksvraag, die concreet geformuleerd is. De achtergrond wordt afdoende toegelicht.
De doelstelling is SMART (specifiek, meetbaar, acceptabel, realistisch en tijdsgebonden) geformuleerd.

Bij WP1-3 is het helder wat de beoogde opbrengsten zijn en voor wie deze zijn bedoeld.
Bij WP 4 is dit zeer algemeen gehouden en kan verdere toelichting hierover meer duidelijkheid verschaffen

1.2 Plan van aanpak
De opzet mbt retrospectieve analyses vanuit bestaande databases lijkt in de huidige situatie binnen een beperkt tijdsinterval het meest haalbare. Het onderscheid tussen de diverse patiëntgroepen WP 1-4 is adequaat om de bijhorende vraagstelling zo goed mogelijk te beantwoorden.

1.3 Haalbaarheid
WP 1-4 lijkt obv de verstrekte informatie haalbaar wat de beschreven dataverzameling betreft.

1.4. Kennisoverdracht en implementatie
De wijze waarop de kennisoverdracht en implementatie zal plaatsvinden staat als dusdanig niet benoemd in het aanvraagdossier.

2.2 Zwakke punten kwaliteit: toelichting naar de aanvragers

Het voorstel bestaat waarschijnlijk uit meerdere werkpakketten. Maak in uw beoordeling per punt duidelijk op welk werkpakket uw commentaar betrekking heeft.

Benoem zwakke punten per onderdeel:

1.1. Probleemstelling en doelstellingen

1.2. Plan van aanpak

1.3. Haalbaarheid

1.4. Kennisoverdracht en implementatie

1.1 Probleemstelling en doelstelling:

WP 1-4:
Enige opmerkingen hierbij:
- Er wordt gesteld dat alle patiënten met nood aan zuurstofsuppletie behandeld worden met dexamethason. In fase I van het ziektebeeld is dit echter niet wenselijk. Hoewel dit slechts een minderheid van de patiënten bij opname betreft, dient hiermee (de ziekteduur) wel rekening te worden gehouden.

- In de aanvraag wordt benoemd dat het verhogen van de steroid dosering , nadat gebleken is dat patiënten niet-responsief zijn op de kuur dexamethason, mogelijk te laat is door escalatie van de pathofysiologische pathways.
Hiervoor bestaan inderdaad sterke argumenten.

Daarom is het onduidelijk waarom in dit onderzoeksvoorstel analyses plaatsvinden bij de patiëntengroep bij wie het ziektebeeld dusdanig ontspoord is dat IC-opname geïndiceerd is.
Het identificeren van de patiëntengroep die voordeel heeft bij vroegtijdige escalatie naar hoge-dosis steroïden ter voorkómen van verdere achteruitgang lijkt hierbij zinvoller.
In de voorgestelde opzet krijgen de patiënten langdurige (>10d) behandeling met steroïden, een deel van hen zelfs na 10d een escalatie naar hogere dosis. Dit leidt tot een verhoogd risico op negatieve effecten zoals bacteriële en schimmelinfecties. Dit is een extra reden om juist vroegtijdig een groep met hyperinflammatie te identificeren, die het meeste baat heeft bij vroegtijdige, intensieve, therapie met hogere dosis steroïden.
Met andere woorden: identificatie ten tijde van opname of in de eerste dagen hierna.
Anders lijkt de doelstelling: "optimal timing of steroids in hospitalized patients" niet behaald te kunnen worden.
- WP-1: de vraagstelling betreft 'dexamethasone-unresponsive patients on the ward' en de interventie betreft IC-patiënten.
Graag toelichting

1.2. Plan van aanpak
- WP 1-4:
Volgens de beschrijving worden uitsluitend analyses bij IC-patiënten verricht. Is dit correct?
Werd er in de deelnemende ziekenhuizen gebruikt gemaakt van HFNC op de verpleegafdelingen?
Zo ja, worden deze meegenomen in de analyses?
Idem voor patiënten met DNR/NIC-beleid en een ernstig ziektebeloop.

1.3. Haalbaarheid
WP 1-4
Het betreft retrospectief onderzoek op data uit bestaande databases. Dit kent uiteraard zijn beperkingen, maar zoals eerder vermeld lijkt dit in de huidige situatie wel het meest haalbare.
Om de haalbaarheid van het project in te schatten is wel bijkomende informatie met betrekking tot de inhoud van de betreffende databases nodig.
- Wat is de inschatting van de patiëntaantallen die gedurende >10 dagen behandeld zijn met steroïden? Betreft dit de genoemde aantallen van resp. 500, 900 en 2400 voor WP-1 tot3? Uitgesplitst in lage vs hoge dosis?
WP-4: werden de te onderzoeken bepalingen routinematig verricht in de deelnemende ziekenhuizen of worden deze verricht op spijtserum?
Indien op spijtserum: is dit aanwezig van alle patiënten? Bepalingen zoals D-dimeer zijn niet altijd betrouwbaar na te bepalen uit spijtserum, zijn deze protocollair verricht in de deelnemende centra?
Is SARS-COV2 RNA bepaling ook protocollair verricht?
- Welke gegevens zijn er momenteel bekend over de diversiteit van behandeling met steroïden in centra waarvan de data in de gebruikte databases zitten?

1.4. Kennisoverdracht en implementatie
hier wordt melding gemaakt van de toevoeging van 3 topklinische ziekenhuizen (7. pagina 9). In de tekst worden alleen OLVG en AZN genoemd.

2.3 Samenvattend oordeel kwaliteit

ZG	G	V	M	O
		X		

Geef een samenvattend oordeel over de kwaliteit van de aanvraag.

Zeer goed: Het voorstel adresseert de kwaliteitscriteria zeer goed, en ik heb eventueel een aantal suggesties voor kleine verbeteringen

Goed: Het voorstel adresseert de kwaliteitscriteria goed, maar een aantal verbeteringen zijn noodzakelijk

Voldoende: In het algemeen zijn de kwaliteitscriteria geadresseerd, maar er zijn significante tekortkomingen die gecorrigeerd dienen te worden

Matig: Het voorstel vertoont tekortkomingen met betrekking tot de kwaliteitscriteria, verbetering is noodzakelijk

Onvoldoende: Het voorstel vertoont ernstige tekortkomingen met betrekking tot de kwaliteitscriteria

3. Relevantiecriteria

Beantwoorden kennishiaat
Het onderzoek levert de urgent benodigde kennis om het in de kennisagenda COVID-19 beschreven kennishiaat te beantwoorden. Geef indien mogelijk aan welke werkpakketten het meest urgent en welke het minst urgent zijn voor het beantwoorden van de kennisvraag.

Impact.
Het onderzoek is van groot belang omdat het gedurende de pandemie, en de nasleep daarvan, impact kan hebben op de behandeling van COVID-19 patiënten; of bijdraagt aan het beter voorbereid zijn op een eventuele volgende pandemie.

Implementeerbare tussenresultaten.
De aanvraag moet overtuigend onderbouwen dat onderzoek op korte termijn leidt tot implementeerbare (tussen)resultaten (tenminste tijdens de pandemie en daarnaast ook voor de lange termijn).

Unieke
positie onderzoeksveld en toegevoegde waarde naast ander onderzoek, er wordt zoveel mogelijk gebruik gemaakt van lopend onderzoek en/of registraties en cohorten

Nieuw onderzoek
Het onderzoek wordt niet al (inter)nationaal uitgevoerd (aanvrager moet dit overtuigend onderbouwen), of de aanvrager moet overtuigend onderbouwen dat onderzoek in Nederland nodig is naast internationaal onderzoek.

Geschikte onderzoeksgroep
Er is een competente onderzoeksgroep/ consortium/ samenwerkingsverband beschikbaar om dit op korte termijn op te pakken. In het consortium is een goede balans tussen academische, STZ en perifere ziekenhuizen en waar van toepassing samenwerking met eerstelijnsorganisaties. Kennishiaat wordt zoveel mogelijk in samenhang opgepakt, door de zorgketen heen.

Voortbouwen op lopend onderzoek en bestaande cohorten en/of databestanden
In deze ronde is in principe alleen financiering beschikbaar voor projecten die voortbouwen op lopend onderzoek en/of gebruik maken, koppelen en doorontwikkelingen van bestaande cohorten en/of databestanden.

Draagvlak
Bij beroepsgroepen en zorginstellingen is voldoende geborgd

Patiëntenparticipatie
Patiëntenparticipatie in het consortium is vereist, als partner vanaf protocol ontwikkeling. Met participatie wordt concreet bedoeld het raadplegen, advies inwinnen, samenwerken en/of laten (mee)beslissen van betrokkenen.

Diversiteit
Aanvragers dienen in de opzet, uitvoering, analyse en rapportage van resultaten onderscheid te maken tussen mannen en vrouwen en andere relevante subgroepen op basis van diversiteit. Indien diversiteit niet relevant is voor het voorgestelde onderzoek, wordt dit voldoende onderbouwd?

4. Samenvattend oordeel relevantie

Legenda: ZR (Zeer relevant), R (Relevant), LR (Laag relevant)

4.1 Sterke punten relevantie: toelichting naar de aanvragers
Het voorstel bestaat waarschijnlijk uit meerdere werkpakketten. Maak in uw beoordeling per punt duidelijk op welk werkpakket uw commentaar betrekking heeft

Benoem sterke punten per onderdeel en benoem duidelijk over werkpakket het gaat:

3.1. Beantwoorden kennishiaat

3.2. Impact

3.3. Implementeerbare tussenresultaten

3.4. Unieke positie

3.5. Nieuw onderzoek

3.6. Geschikte onderzoeksgroep

3.7. Voortbouwen op lopend onderzoek en bestaande cohorten en/of databestanden

3.8. Draagvlak

3.9. Patientenparticipatie

3.10. Diversiteit

3.1. Beantwoorden kennishiaat
Er wordt gestreefd om meer duidelijkheid te verschaffen over de meerwaarde van hogere dosis steroiden bij ernstig zieke COVID-19 patiënten

3.2. Impact
Het onderzoek is in potentie van groot belang omdat het gedurende de pandemie, en de nasleep daarvan, impact kan hebben op de behandeling van COVID-19 patiënten; of bijdraagt aan het beter voorbereid zijn op een eventuele volgende pandemie.

3.3. Implementeerbare tussenresultaten
De aanvraag is redelijk adequaat onderbouwd dat het onderzoek op korte termijn leidt tot implementeerbare (tussen)resultaten (tenminste tijdens de pandemie en daarnaast ook voor de lange termijn

3.4. Unieke positie
er wordt zoveel mogelijk gebruik gemaakt van lopend onderzoek en/of registraties en cohorten

3.5. Nieuw onderzoek
zie opmerkingen bij 4.2.3.5

3.6. Geschikte onderzoeksgroep
Er is een competente onderzoeksgroep/ consortium/ samenwerkingsverband beschikbaar om dit op korte termijn op te pakken. In het consortium is een goede balans tussen academische, STZ en perifere ziekenhuizen

3.7. Voortbouwen op lopend onderzoek en bestaande cohorten en/of databestanden
Hieraan wordt voldaan.

3.8. Draagvlak
Bij beroepsgroepen en zorginstellingen is voldoende geborgd

3.9. Patientenparticipatie
Hiervan wordt geen melding gemaakt in de aanvraag.
3.10. Diversiteit
Hiervan wordt geen melding gemaakt in de aanvraag.

4.2 Zwakke punten relevantie: toelichting naar de aanvragers
Het voorstel bestaat waarschijnlijk uit meerdere werkpakketten. Maak in uw beoordeling per punt duidelijk op welk werkpakket uw commentaar betrekking heeft

Benoem zwakke punten per onderdeel:

3.1. Beantwoorden kennishiaat

3.2. Impact

3.3. Implementeerbare tussenresultaten

3.4. Unieke positie

3.5. Nieuw onderzoek

3.6. Geschikte onderzoeksgroep

3.7. Voortbouwen op lopend onderzoek en bestaande cohorten en/of databestanden

3.8. Draagvlak

3.9. Patientenparticipatie

3.10. Diversiteit

3.1. Beantwoorden kennishiaat
Door te focussen op patiënten die reeds een gevorderd ziektebeloop hebben, wordt de kans gemist om de patiëntengroep te identificeren die baat heeft bij vroegtijdige behandeling met hogere dosis steroïden om verdere verslechtering te voorkómen.

3.2. Impact
De impact kan wellicht vergroot worden door het identificeren van de groep benoemd in 3.1
Tevens is de impact van het huidige protocol nu onvoldoende in te schatten gezien het onduidelijk is hoeveel patiënten de te onderzoeken therapie ondergaan hebben. In veel ziekenhuizen is pas laat in de eerste golf of vanaf de tweede golf gestart met behandeling met steroiden, waardoor de groep beperkt is. Voortgezette (>10d) behandeling en behandeling met hogere dosis steroiden is in een nog kleinere groep toegepast.

3.3. Implementeerbare tussenresultaten
Afhankelijk van de opmerkingen zoals beschreven in 3.1 en 3.2

3.4. Unieke positie
positie onderzoeksveld en toegevoegde waarde naast ander onderzoek wordt onvoldoende toegelicht.

3.5. Nieuw onderzoek
Er wordt onvoldoende onderbouwd of het onderzoek niet al (inter)nationaal uitgevoerd wordt, of dat onderzoek in Nederland nodig is naast internationaal onderzoek.

3.6. Geschikte onderzoeksgroep
geen twijfels hierover.

3.7. Voortbouwen op lopend onderzoek en bestaande cohorten en/of databestanden
Hieraan wordt voldaan.

3.8. Draagvlak
geen opmerkingen hierover

3.9. Patientenparticipatie
Hiervan wordt geen melding gemaakt in de aanvraag.

3.10. Diversiteit
Hiervan wordt geen melding gemaakt in de aanvraag.

4.3 Samenvattend oordeel relevantie

ZR	R	LR
	X	

Geef een samenvattend relevantie oordeel

5. Begroting

Legenda: TH (Te hoog), R (reëel), TL (te laag)

5.1 Begroting

TH	R	TL
	X	

In hoeverre is het budget realistisch gezien de geplande activiteiten? Is de toelichting op de begroting voldoende helder?

De begroting lijkt realistisch, geen opmerkingen hierover.

Subsidieprogramma / Subsidy programme	:	**COVID-19 Programma**
Dossiernummer / Dossier number	:	**50-56300-98-2115**
Aanvrager / applicant	:	**Dr. H. Endeman**
Projecttitel / Project title	:	**Optimal dosing and timing of steroids in hospitalized patients with COVID-19**
Beoordelingscode / Assessment code	:	**B.2021.013BE**

1. Kwaliteitscriteria

Doelstelling en vraag- of taakstelling

Denk daarbij aan:
- Helderheid, concreetheid vraag- of taakstelling en doelstelling. Is de doelstelling SMART (specifiek, meetbaar, acceptabel, realistisch en tijdsgebonden) geformuleerd?
- Is helder wat de beoogde opbrengsten zijn en voor wie deze zijn bedoeld?

Plan van aanpak

Denk daarbij aan:
- Theoretische of empirische onderbouwing
- Adequaatheid van plan van aanpak voor de vraagstelling/doelstelling
- Geschiktheid van gekozen methoden/analyses (design, studiepopulatie, data-bronnen)
- Is er een heldere powerberekening toegevoegd, indien van toepassing?

Haalbaarheid

Denk daarbij aan:
- Is het een realistische verwachting dat het project snel kan starten?
- Is het onderzoek haalbaar in de huidige pandemiesituatie?
- Is de recruteringsstrategie en inclusie verwachting realistisch?
- Is de beschikbaarheid van faciliteiten/mensen realistisch?
- Zijn de verwachte resultaten, fasering en tijdsplanning realistisch?

Kennisoverdracht en implementatie

Denk daarbij aan:
- goede mix van activiteiten voor kennisoverdracht en/of bestendiging;
- mate waarin de betrokkenheid van gebruikers en doelgroepen (inclusief patiëntenvertegenwoordigers) aandacht krijgt;
- is de implementatiestrategie duidelijk?

2. Samenvattend oordeel kwaliteit

Legenda: ZG (zeer goed), G (goed), V (voldoende), M (matig), O (onvoldoende)

2.1 Sterke punten kwaliteit: toelichting naar de aanvragers

Het voorstel bestaat waarschijnlijk uit meerdere werkpakketten. Maak in uw beoordeling per punt duidelijk op welk werkpakket uw commentaar betrekking heeft.

Benoem sterke punten per onderdeel:

1.1. Probleemstelling en doelstellingen

1.2. Plan van aanpak

1.3. Haalbaarheid

1.4. Kennisoverdracht en implementatie

1.1 Duidelijke probleemstelling.

1.2 duidelijk plan van aanpak, waarbij gebruik wordt gemaakt van bestaande data en samenwerking met meerdere partners

1.3

1.4

2.2 Zwakke punten kwaliteit: toelichting naar de aanvragers

Het voorstel bestaat waarschijnlijk uit meerdere werkpakketten. Maak in uw beoordeling per punt duidelijk op welk werkpakket uw commentaar betrekking heeft.

Benoem zwakke punten per onderdeel:

1.1. Probleemstelling en doelstellingen

1.2. Plan van aanpak

1.3. Haalbaarheid

1.4. Kennisoverdracht en implementatie

1.2
Ik vind het jammer dat er in dit plan alleen gekeken wordt naar de klinische uitkomsten, zoals bij wp 1 Uitkomstmaten: 28 dagen overleving, 28 dagen ziekenhuisvrije dagen, 28 dagen invasieve mechanische beademing. Kunnen niet ook andere lange termijn uitkomsten, het ontwikkelen van welke morbiditeiten, zoals het ontwikkelen van long covid worden meegenomen Daarnaast is er in het onderzoek geen aandacht voor de ervaring van de patiënt, bijvoorbeeld kwaliteit van leven /impact op de patiënt zelf, ik kan me voorstellen dat ook bij het tijdstip dat de patiënt de steroïden krijgt hier invloed op heeft.

Als dit niet verkregen kan worden uit de gebruikte data is de toevoeging van een raadpleging onder deze patiënten wellicht nog een goede toevoeging. Hierbij zou ook het aspect samen beslissen - kunnen worden meegenomen.

1.4 Ik vind het implementatieplan nog niet duidelijk beschreven. Alle ziekenhuizen en partners zijn duidelijk zeer capabel, maar wanneer kunnen eerste resultaten gepubliceerd worden en ingezet worden en hoe wordt dit beschikbaar gemaakt voor andere ziekenhuizen? Voordat deze kunnen worden opgenomen in richtlijn Covid

2.3 Samenvattend oordeel kwaliteit

ZG	G	V	M	O
	X			

Geef een samenvattend oordeel over de kwaliteit van de aanvraag.

Zeer goed: Het voorstel adresseert de kwaliteitscriteria zeer goed, en ik heb eventueel een aantal suggesties voor kleine verbeteringen

Goed: Het voorstel adresseert de kwaliteitscriteria goed, maar een aantal verbeteringen zijn noodzakelijk

Voldoende: In het algemeen zijn de kwaliteitscriteria geadresseerd, maar er zijn significante tekortkomingen die gecorrigeerd dienen te worden

Matig: Het voorstel vertoont tekortkomingen met betrekking tot de kwaliteitscriteria, verbetering is noodzakelijk

Onvoldoende: Het voorstel vertoont ernstige tekortkomingen met betrekking tot de kwaliteitscriteria

3. Relevantiecriteria

Beantwoorden kennishiaat
Het onderzoek levert de urgent benodigde kennis om het in de kennisagenda COVID-19 beschreven kennishiaat te beantwoorden. Geef indien mogelijk aan welke werkpakketten het meest urgent en welke het minst urgent zijn voor het beantwoorden van de kennisvraag.

Impact.
Het onderzoek is van groot belang omdat het gedurende de pandemie, en de nasleep daarvan, impact kan hebben op de behandeling van COVID-19 patiënten; of bijdraagt aan het beter voorbereid zijn op een eventuele volgende pandemie.

Implementeerbare tussenresultaten.
De aanvraag moet overtuigend onderbouwen dat onderzoek op korte termijn leidt tot implementeerbare (tussen)resultaten (tenminste tijdens de pandemie en daarnaast ook voor de lange termijn).

Unieke
positie onderzoeksveld en toegevoegde waarde naast ander onderzoek, er wordt zoveel mogelijk gebruik gemaakt van lopend onderzoek en/of registraties en cohorten

Nieuw onderzoek
Het onderzoek wordt niet al (inter)nationaal uitgevoerd (aanvrager moet dit overtuigend onderbouwen), of de aanvrager moet overtuigend onderbouwen dat onderzoek in Nederland nodig is naast internationaal onderzoek.

Geschikte onderzoeksgroep
Er is een competente onderzoeksgroep/ consortium/ samenwerkingsverband beschikbaar om dit op korte termijn op te pakken. In het consortium is een goede balans tussen academische, STZ en perifere ziekenhuizen en waar van toepassing samenwerking met eerstelijnsorganisaties. Kennishiaat wordt zoveel mogelijk in samenhang opgepakt, door de zorgketen heen.

Voortbouwen op lopend onderzoek en bestaande cohorten en/of databestanden
In deze ronde is in principe alleen financiering beschikbaar voor projecten die voortbouwen op lopend onderzoek en/of gebruik maken, koppelen en doorontwikkelingen van bestaande cohorten en/of databestanden.

Draagvlak
Bij beroepsgroepen en zorginstellingen is voldoende geborgd

Patiëntenparticipatie
Patiëntenparticipatie in het consortium is vereist, als partner vanaf protocol ontwikkeling. Met participatie wordt concreet bedoeld het raadplegen, advies inwinnen, samenwerken en/of laten (mee)beslissen van betrokkenen.

Diversiteit
Aanvragers dienen in de opzet, uitvoering, analyse en rapportage van resultaten onderscheid te maken tussen mannen en vrouwen en andere relevante subgroepen op basis van diversiteit. Indien diversiteit niet relevant is voor het voorgestelde onderzoek, wordt dit voldoende onderbouwd?

4. Samenvattend oordeel relevantie

Legenda: ZR (Zeer relevant), R (Relevant), LR (Laag relevant)

4.1 Sterke punten relevantie: toelichting naar de aanvragers

Het voorstel bestaat waarschijnlijk uit meerdere werkpakketten. Maak in uw beoordeling per punt duidelijk op welk werkpakket uw commentaar betrekking heeft

Benoem sterke punten per onderdeel en benoem duidelijk over werkpakket het gaat:

3.1. Beantwoorden kennishiaat

3.2. Impact

3.3. Implementeerbare tussenresultaten

3.4. Unieke positie

3.5. Nieuw onderzoek

3.6. Geschikte onderzoeksgroep

3.7. Voortbouwen op lopend onderzoek en bestaande cohorten en/of databestanden

3.8. Draagvlak

3.9. Patientenparticipatie

3.10. Diversiteit

3.1. Beantwoorden kennishiaat - ik denk het onderzoek voldoet aan het beantwoorden van de kennisvraag waar een grote behoefte aan is.

3.2. Impact
3.6. Geschikte onderzoeksgroep
Relevante onderzoekspopulatie en duidelijke behoefte aan een goede behandeling voor deze groep patienten.

3.8. Draagvlak
Er is terecht een groot draagvlak voor deze onderzoeksvraag. Ook patienten willen graag weten wat de juiste behandeling en timing is

3.9
Patientenparticipatie
2 goed aangeschreven/capabele patiëntenorganisaties zijn betrokken bij deze aanvraag, zowel bij het design, analyse en interpretatie van het onderzoek. Zij hebben ook ervaring met dit soort onderzoeken.

4.2 Zwakke punten relevantie: toelichting naar de aanvragers
Het voorstel bestaat waarschijnlijk uit meerdere werkpakketten. Maak in uw beoordeling per punt duidelijk op welk werkpakket uw commentaar betrekking heeft

Benoem zwakke punten per onderdeel:

3.1. Beantwoorden kennishiaat

3.2. Impact

3.3. Implementeerbare tussenresultaten

3.4. Unieke positie

3.5. Nieuw onderzoek

3.6. Geschikte onderzoeksgroep

3.7. Voortbouwen op lopend onderzoek en bestaande cohorten en/of databestanden

3.8. Draagvlak

3.9. Patientenparticipatie

3.10. Diversiteit

3.3. Implementeerbare tussenresultaten

Ik vind dit onvoldoende beschreven, kunt u hier meer over aangeven?

3.6. Geschikte onderzoeksgroep
Is voor de WP4b study de grootte van 50 voldoende om diversiteit te kunnen vinden?

3.8. Draagvlak
Er is terecht een groot draagvlak voor deze onderzoeksvraag. Ook patiënten willen graag weten wat de juiste behandeling en timing is, in de uitkomstmaten mis ik nog wel relevante patiënten uitkomsten, zoals lange termijn effecten, morbiditeit, en wat het (moment van het) krijgen van steriÖde voor de patiënt heeft betekend.

3.9
Patientenparticipatie

Ondanks de betrokkenheid van 2 patiëntenorganisaties is er geen budget voor beschikbaar in de begroting.

Beide patiëntenorganisaties zijn gericht op de opname op de IC, is het voor deze onderzoek aanvraag niet ook relevant om patiënten vanuit de aandoeningen te betrekken, zoals longpatiënten?

Ik mis de betrokkenheid van patiënten die ook in deze situatie zijn geweest. Hoe is hierin voorzien?

4.3 Samenvattend oordeel relevantie

ZR	R	LR
	X	

Geef een samenvattend relevantie oordeel

5. Begroting

Legenda: TH (Te hoog), R (reëel), TL (te laag)

5.1 Begroting

TH	R	TL
		X

In hoeverre is het budget realistisch gezien de geplande activiteiten? Is de toelichting op de begroting voldoende helder?

Er is geen budget opgenomen voor patiëntenparticipatie en naast de kosten voor het publiceren(5000) is er niets opgenomen voor het verspreiden van deze kennis.

Subsidieprogramma / Subsidy programme	:	**COVID-19 Programma**
Dossiernummer / Dossier number	:	**50-56300-98-2115**
Aanvrager / applicant	:	**Dr. H. Endeman**
Projecttitel / Project title	:	**Optimal dosing and timing of steroids in hospitalized patients with COVID-19**
Beoordelingscode / Assessment code	:	**B.2021.013BF**

1. Kwaliteitscriteria

Doelstelling en vraag- of taakstelling

Denk daarbij aan:
- Helderheid, concreetheid vraag- of taakstelling en doelstelling. Is de doelstelling SMART (specifiek, meetbaar, acceptabel, realistisch en tijdsgebonden) geformuleerd?
- Is helder wat de beoogde opbrengsten zijn en voor wie deze zijn bedoeld?

Plan van aanpak

Denk daarbij aan:
- Theoretische of empirische onderbouwing
- Adequaatheid van plan van aanpak voor de vraagstelling/doelstelling
- Geschiktheid van gekozen methoden/analyses (design, studiepopulatie, data-bronnen)
- Is er een heldere powerberekening toegevoegd, indien van toepassing?

Haalbaarheid

Denk daarbij aan:
- Is het een realistische verwachting dat het project snel kan starten?
- Is het onderzoek haalbaar in de huidige pandemiesituatie?
- Is de recruteringsstrategie en inclusie verwachting realistisch?
- Is de beschikbaarheid van faciliteiten/mensen realistisch?
- Zijn de verwachte resultaten, fasering en tijdsplanning realistisch?

Kennisoverdracht en implementatie

Denk daarbij aan:
- goede mix van activiteiten voor kennisoverdracht en/of bestendiging;
- mate waarin de betrokkenheid van gebruikers en doelgroepen (inclusief patiëntenvertegenwoordigers) aandacht krijgt;
- is de implementatiestrategie duidelijk?

2. Samenvattend oordeel kwaliteit

Legenda: ZG (zeer goed), G (goed), V (voldoende), M (matig), O (onvoldoende)

2.1 Sterke punten kwaliteit: toelichting naar de aanvragers

Het voorstel bestaat waarschijnlijk uit meerdere werkpakketten. Maak in uw beoordeling per punt duidelijk op welk werkpakket uw commentaar betrekking heeft.

Benoem sterke punten per onderdeel:

1.1. Probleemstelling en doelstellingen

1.2. Plan van aanpak

1.3. Haalbaarheid

1.4. Kennisoverdracht en implementatie

1.1 Probleemstelling en doelstellingen zijn duidelijk, recent is een letter to the editor gepubliceerd waarin het probleem ook duidelijk is verwoord, mogelijke oplossingsrichtingen zijn benoemd en de oproep gedaan tot voldoende gepowerd onderzoek. Zie bijlage 'Letter to the editor 82297-316469-1-PB.pdf'.
Mogelijk kunnen de auteurs dit nog in de inleiding verwerken.

1.2 Plan van aanpak is duidelijk, alhoewel er geen cijfers zijn van de te verwachten inclusies in WP's 1-3. Uit een Japanse studie lijkt te blijken dat het percentage steroid resistentie beperkt is (https://doi.org/10.1016/j.resinv.2021.05.007). Dezelfde Japanse studie laat zien dat verlenging van de standaard 10-dagen corticosteroid therapie mogelijk het percentage steroid resistentie kan verlagen. Waarom dit niet als arm meegenomen?

1.3 Haalbaarheid is afhankelijk van de gewenste inclusie (zie kommentaar bij 1.2) en power om een uitspraak te doen.

1.4 Als er resultaten zijn, kan overdracht en implementatie snel plaatsvinden.

2.2 Zwakke punten kwaliteit: toelichting naar de aanvragers

Het voorstel bestaat waarschijnlijk uit meerdere werkpakketten. Maak in uw beoordeling per punt duidelijk op welk werkpakket uw commentaar betrekking heeft.

Benoem zwakke punten per onderdeel:

1.1. Probleemstelling en doelstellingen

1.2. Plan van aanpak

1.3. Haalbaarheid

1.4. Kennisoverdracht en implementatie

1.1 onduidelijk wat de gewenste inclusie is per WP

1.2 geen opm.

1.3 Hangt samen met 1.1 en het aantal steroid resistente patienten per WP. Data ontbreken.

1.4 geen opm.

2.3 Samenvattend oordeel kwaliteit

ZG	G	V	M	O
		X		

Geef een samenvattend oordeel over de kwaliteit van de aanvraag.

Zeer goed: Het voorstel adresseert de kwaliteitscriteria zeer goed, en ik heb eventueel een aantal suggesties voor kleine verbeteringen

Goed: Het voorstel adresseert de kwaliteitscriteria goed, maar een aantal verbeteringen zijn noodzakelijk

Voldoende: In het algemeen zijn de kwaliteitscriteria geadresseerd, maar er zijn significante tekortkomingen die gecorrigeerd dienen te worden

Matig: Het voorstel vertoont tekortkomingen met betrekking tot de kwaliteitscriteria, verbetering is noodzakelijk

Onvoldoende: Het voorstel vertoont ernstige tekortkomingen met betrekking tot de kwaliteitscriteria

3. Relevantiecriteria

Beantwoorden kennishiaat
Het onderzoek levert de urgent benodigde kennis om het in de kennisagenda COVID-19 beschreven kennishiaat te beantwoorden. Geef indien mogelijk aan welke werkpakketten het meest urgent en welke het minst urgent zijn voor het beantwoorden van de kennisvraag.

Impact.
Het onderzoek is van groot belang omdat het gedurende de pandemie, en de nasleep daarvan, impact kan hebben op de behandeling van COVID-19 patiënten; of bijdraagt aan het beter voorbereid zijn op een eventuele volgende pandemie.

Implementeerbare tussenresultaten.
De aanvraag moet overtuigend onderbouwen dat onderzoek op korte termijn leidt tot implementeerbare (tussen)resultaten (tenminste tijdens de pandemie en daarnaast ook voor de lange termijn).

Unieke
positie onderzoeksveld en toegevoegde waarde naast ander onderzoek, er wordt zoveel mogelijk gebruik gemaakt van lopend onderzoek en/of registraties en cohorten

Nieuw onderzoek
Het onderzoek wordt niet al (inter)nationaal uitgevoerd (aanvrager moet dit overtuigend onderbouwen), of de aanvrager moet overtuigend onderbouwen dat onderzoek in Nederland nodig is naast internationaal onderzoek.

Geschikte onderzoeksgroep
Er is een competente onderzoeksgroep/ consortium/ samenwerkingsverband beschikbaar om dit op korte termijn op te pakken. In het consortium is een goede balans tussen academische, STZ en perifere ziekenhuizen en waar van toepassing samenwerking met eerstelijnsorganisaties. Kennishiaat wordt zoveel mogelijk in samenhang opgepakt, door de zorgketen heen.

Voortbouwen op lopend onderzoek en bestaande cohorten en/of databestanden
In deze ronde is in principe alleen financiering beschikbaar voor projecten die voortbouwen op lopend onderzoek en/of gebruik maken, koppelen en doorontwikkelingen van bestaande cohorten en/of databestanden.

Draagvlak
Bij beroepsgroepen en zorginstellingen is voldoende geborgd

Patiëntenparticipatie
Patiëntenparticipatie in het consortium is vereist, als partner vanaf protocol ontwikkeling. Met participatie wordt concreet bedoeld het raadplegen, advies inwinnen, samenwerken en/of laten (mee)beslissen van betrokkenen.

Diversiteit
Aanvragers dienen in de opzet, uitvoering, analyse en rapportage van resultaten onderscheid te maken tussen mannen en vrouwen en andere relevante subgroepen op basis van diversiteit. Indien diversiteit niet relevant is voor het voorgestelde onderzoek, wordt dit voldoende onderbouwd?

4. Samenvattend oordeel relevantie

Legenda: ZR (Zeer relevant), R (Relevant), LR (Laag relevant)

4.1 Sterke punten relevantie: toelichting naar de aanvragers
Het voorstel bestaat waarschijnlijk uit meerdere werkpakketten. Maak in uw beoordeling per punt duidelijk op welk werkpakket uw commentaar betrekking heeft

Benoem sterke punten per onderdeel en benoem duidelijk over werkpakket het gaat:

3.1. Beantwoorden kennishiaat

3.2. Impact

3.3. Implementeerbare tussenresultaten

3.4. Unieke positie

3.5. Nieuw onderzoek

3.6. Geschikte onderzoeksgroep

3.7. Voortbouwen op lopend onderzoek en bestaande cohorten en/of databestanden

3.8. Draagvlak

3.9. Patientenparticipatie

3.10. Diversiteit

3.1 In principe kan met deze studie het kennishiaat ingevuld worden.

3.2 De impact kan groot zijn, zie eerdere opm over LttE.

3.3 Tussenresultaten kunnen direct leiden tot implementatie, zal wel effect hebben op controlegroep.

3.4 De combinatie van EMC en Amsterdam UMC is voldoende groot om de studie succesvol te laten zijn. Expertise staat buiten kijf.

3.5 Het is geen nieuw concept, de vraag naar deze studie is gesteld in eerder genoemde LttE.

3.6 Onderzoeksgroep is voldoende gekwalificeerd.

3.7 Er wordt gebruik gemaakt van bestaande biobanken.

3.8 Draagvlak lijkt voldoende gezien de door de FMS geformuleerde kennishiaat.

3.9 Is in orde.

3.10 Hier wordt niets over gezegd, maar er worden geen groepen geexcludeerd.

4.2 Zwakke punten relevantie: toelichting naar de aanvragers

Het voorstel bestaat waarschijnlijk uit meerdere werkpakketten. Maak in uw beoordeling per punt duidelijk op welk werkpakket uw commentaar betrekking heeft

Benoem zwakke punten per onderdeel:

3.1. Beantwoorden kennishiaat

3.2. Impact

3.3. Implementeerbare tussenresultaten

3.4. Unieke positie

3.5. Nieuw onderzoek

3.6. Geschikte onderzoeksgroep

3.7. Voortbouwen op lopend onderzoek en bestaande cohorten en/of databestanden

3.8. Draagvlak

3.9. Patientenparticipatie

3.10. Diversiteit

3.1 Zwak punt blijft de inclusie en voldoende power gezien het % falen op steroid therapie.

3.2 geen opm.

3.3 geen opm.

3.4 Waarom niet ook andere (acad) centra benaderd om de inclusie te versnellen? De vraag is urgent.

3.5 geen opm.

3.6 geen opm.

3.7 geen opm.

3.8 zie 3.4

3.9 geen opm.

3.10 misschien opmerking maken over in/exclusie?

4.3 Samenvattend oordeel relevantie

ZR	R	LR
	X	

Geef een samenvattend relevantie oordeel

5. Begroting

Legenda: TH (Te hoog), R (reëel), TL (te laag)

5.1 Begroting

TH	R	TL
	X	

In hoeverre is het budget realistisch gezien de geplande activiteiten? Is de toelichting op de begroting voldoende helder?

Begroting lijkt me reëel en zelfs aan de lage kant, 3 promovendi en kosten voor het biomarker onderzoek. Het valt me op dat de kosten voor begeleiding niet expliciet zijn benoemd. Het kunnen afronden van de promotie na 2 jaar wordt door de instituten gegarandeerd. Dit is een positieve incentive.

Subsidieprogramma / Subsidy programme	:	**COVID-19 Programma**
Dossiernummer / Dossier number	:	**50-56300-98-2115**
Aanvrager / applicant	:	**Dr. H. Endeman**
Projecttitel / Project title	:	**Optimal dosing and timing of steroids in hospitalized patients with COVID-19**
Beoordelingscode / Assessment code	:	**B.2021.013C1**

1. Kwaliteitscriteria

Doelstelling en vraag- of taakstelling

Denk daarbij aan:

- Helderheid, concreetheid vraag- of taakstelling en doelstelling. Is de doelstelling SMART (specifiek, meetbaar, acceptabel, realistisch en tijdsgebonden) geformuleerd?
- Is helder wat de beoogde opbrengsten zijn en voor wie deze zijn bedoeld?

Plan van aanpak

Denk daarbij aan:

- Theoretische of empirische onderbouwing
- Adequaatheid van plan van aanpak voor de vraagstelling/doelstelling
- Geschiktheid van gekozen methoden/analyses (design, studiepopulatie, data-bronnen)
- Is er een heldere powerberekening toegevoegd, indien van toepassing?

Haalbaarheid

Denk daarbij aan:

- Is het een realistische verwachting dat het project snel kan starten?
- Is het onderzoek haalbaar in de huidige pandemiesituatie?
- Is de recruteringsstrategie en inclusie verwachting realistisch?
- Is de beschikbaarheid van faciliteiten/mensen realistisch?
- Zijn de verwachte resultaten, fasering en tijdsplanning realistisch?

Kennisoverdracht en implementatie

Denk daarbij aan:

- goede mix van activiteiten voor kennisoverdracht en/of bestendiging;
- mate waarin de betrokkenheid van gebruikers en doelgroepen (inclusief patiëntenvertegenwoordigers) aandacht krijgt;
- is de implementatiestrategie duidelijk?

2. Samenvattend oordeel kwaliteit

Legenda: ZG (zeer goed), G (goed), V (voldoende), M (matig), O (onvoldoende)

2.1 Sterke punten kwaliteit: toelichting naar de aanvragers

Het voorstel bestaat waarschijnlijk uit meerdere werkpakketten. Maak in uw beoordeling per punt duidelijk op welk werkpakket uw commentaar betrekking heeft.

Benoem sterke punten per onderdeel:

1.1. Probleemstelling en doelstellingen

1.2. Plan van aanpak

1.3. Haalbaarheid

1.4. Kennisoverdracht en implementatie

1.1 Het probleem is herkenbaar. Patienten die niet op de igestelde behandeling met Dexamethason reageren. Eigen ervaring in de eerste golf (zonder Dexamethason) laat zien dat hoge dosering Methylprednisolon effectief is(retrospectief)
1.2 Voor de snelheid om deze vraag te beantwoorden zijn retrospectieve data relevant. Aangezien de timing van het starten van hoge dosering steroiden niet uniform is heb ik mijn twijfels of er uit de ruis nog signaal te halen is.
1.3 De beschreven opzet met retrospectieve data lijkt me haalbaar.
1.4 Implementatie is eenvoudig

2.2 Zwakke punten kwaliteit: toelichting naar de aanvragers

Het voorstel bestaat waarschijnlijk uit meerdere werkpakketten. Maak in uw beoordeling per punt duidelijk op welk werkpakket uw commentaar betrekking heeft.

Benoem zwakke punten per onderdeel:

1.1. Probleemstelling en doelstellingen

1.2. Plan van aanpak

1.3. Haalbaarheid

1.4. Kennisoverdracht en implementatie

zie vorige beoordeling

2.3 Samenvattend oordeel kwaliteit

ZG	G	V	M	O
		X		

Geef een samenvattend oordeel over de kwaliteit van de aanvraag.

Zeer goed: Het voorstel adresseert de kwaliteitscriteria zeer goed, en ik heb eventueel een aantal suggesties voor kleine verbeteringen

Goed: Het voorstel adresseert de kwaliteitscriteria goed, maar een aantal verbeteringen zijn noodzakelijk

Voldoende: In het algemeen zijn de kwaliteitscriteria geadresseerd, maar er zijn significante tekortkomingen die gecorrigeerd dienen te worden

Matig: Het voorstel vertoont tekortkomingen met betrekking tot de kwaliteitscriteria, verbetering is noodzakelijk

Onvoldoende: Het voorstel vertoont ernstige tekortkomingen met betrekking tot de kwaliteitscriteria

3. Relevantiecriteria

Beantwoorden kennishiaat
Het onderzoek levert de urgent benodigde kennis om het in de kennisagenda COVID-19 beschreven kennishiaat te beantwoorden. Geef indien mogelijk aan welke werkpakketten het meest urgent en welke het minst urgent zijn voor het beantwoorden van de kennisvraag.

Impact.
Het onderzoek is van groot belang omdat het gedurende de pandemie, en de nasleep daarvan, impact kan hebben op de behandeling van COVID-19 patiënten; of bijdraagt aan het beter voorbereid zijn op een eventuele volgende pandemie.

Implementeerbare tussenresultaten.
De aanvraag moet overtuigend onderbouwen dat onderzoek op korte termijn leidt tot implementeerbare (tussen)resultaten (tenminste tijdens de pandemie en daarnaast ook voor de lange termijn).

Unieke
positie onderzoeksveld en toegevoegde waarde naast ander onderzoek, er wordt zoveel mogelijk gebruik gemaakt van lopend onderzoek en/of registraties en cohorten

Nieuw onderzoek
Het onderzoek wordt niet al (inter)nationaal uitgevoerd (aanvrager moet dit overtuigend onderbouwen), of de aanvrager moet overtuigend onderbouwen dat onderzoek in Nederland nodig is naast internationaal onderzoek.

Geschikte onderzoeksgroep
Er is een competente onderzoeksgroep/ consortium/ samenwerkingsverband beschikbaar om dit op korte termijn op te pakken. In het consortium is een goede balans tussen academische, STZ en perifere ziekenhuizen en waar van toepassing samenwerking met eerstelijnsorganisaties. Kennishiaat wordt zoveel mogelijk in samenhang opgepakt, door de zorgketen heen.

Voortbouwen op lopend onderzoek en bestaande cohorten en/of databestanden
In deze ronde is in principe alleen financiering beschikbaar voor projecten die voortbouwen op lopend onderzoek en/of gebruik maken, koppelen en doorontwikkelingen van bestaande cohorten en/of databestanden.

Draagvlak
Bij beroepsgroepen en zorginstellingen is voldoende geborgd

Patiëntenparticipatie
Patiëntenparticipatie in het consortium is vereist, als partner vanaf protocol ontwikkeling. Met participatie wordt concreet bedoeld het raadplegen, advies inwinnen, samenwerken en/of laten (mee)beslissen van betrokkenen.

Diversiteit
Aanvragers dienen in de opzet, uitvoering, analyse en rapportage van resultaten onderscheid te maken tussen mannen en vrouwen en andere relevante subgroepen op basis van diversiteit. Indien diversiteit niet relevant is voor het voorgestelde onderzoek, wordt dit voldoende onderbouwd?

4. Samenvattend oordeel relevantie

Legenda: ZR (Zeer relevant), R (Relevant), LR (Laag relevant)

4.1 Sterke punten relevantie: toelichting naar de aanvragers

Het voorstel bestaat waarschijnlijk uit meerdere werkpakketten. Maak in uw beoordeling per punt duidelijk op welk werkpakket uw commentaar betrekking heeft

Benoem sterke punten per onderdeel en benoem duidelijk over werkpakket het gaat:

3.1. Beantwoorden kennishiaat

3.2. Impact

3.3. Implementeerbare tussenresultaten

3.4. Unieke positie

3.5. Nieuw onderzoek

3.6. Geschikte onderzoeksgroep

3.7. Voortbouwen op lopend onderzoek en bestaande cohorten en/of databestanden

3.8. Draagvlak

3.9. Patientenparticipatie

3.10. Diversiteit

De vraagstelling is zeker relevant. Ik betwijfel of met deze opzet een antwoord op de vraag gegeven kan worden. De onderzoeksgroep bevat voldoende kennis. De patientenpopulat.ie lijkt me groot genoeg om de de vraag te beantwoorden

4.2 Zwakke punten relevantie: toelichting naar de aanvragers

Het voorstel bestaat waarschijnlijk uit meerdere werkpakketten. Maak in uw beoordeling per punt duidelijk op welk werkpakket uw commentaar betrekking heeft

Benoem zwakke punten per onderdeel:

3.1. Beantwoorden kennishiaat

3.2. Impact

3.3. Implementeerbare tussenresultaten

3.4. Unieke positie

3.5. Nieuw onderzoek

3.6. Geschikte onderzoeksgroep

3.7. Voortbouwen op lopend onderzoek en bestaande cohorten en/of databestanden

3.8. Draagvlak

3.9. Patientenparticipatie

3.10. Diversiteit

vide supra

4.3 Samenvattend oordeel relevantie

ZR	R	LR
X		

Geef een samenvattend relevantie oordeel

5. Begroting

Legenda: TH (Te hoog), R (reëel), TL (te laag)

5.1 Begroting

TH	R	TL
	X	

In hoeverre is het budget realistisch gezien de geplande activiteiten? Is de toelichting op de begroting voldoende helder?

Kan ik niet beoordelen

Subsidieprogramma / Subsidy programme	:	**COVID-19 Programma**
Dossiernummer / Dossier number	:	**50-56300-98-2115**
Aanvrager / applicant	:	**Dr. H. Endeman**
Projecttitel / Project title	:	**Optimal dosing and timing of steroids in hospitalized patients with COVID-19**
Beoordelingscode / Assessment code	:	**B.2021.013C2**

1. Kwaliteitscriteria

Doelstelling en vraag- of taakstelling

Denk daarbij aan:
- Helderheid, concreetheid vraag- of taakstelling en doelstelling. Is de doelstelling SMART (specifiek, meetbaar, acceptabel, realistisch en tijdsgebonden) geformuleerd?
- Is helder wat de beoogde opbrengsten zijn en voor wie deze zijn bedoeld?

Plan van aanpak

Denk daarbij aan:
- Theoretische of empirische onderbouwing
- Adequaatheid van plan van aanpak voor de vraagstelling/doelstelling
- Geschiktheid van gekozen methoden/analyses (design, studiepopulatie, data-bronnen)
- Is er een heldere powerberekening toegevoegd, indien van toepassing?

Haalbaarheid

Denk daarbij aan:
- Is het een realistische verwachting dat het project snel kan starten?
- Is het onderzoek haalbaar in de huidige pandemiesituatie?
- Is de recruteringsstrategie en inclusie verwachting realistisch?
- Is de beschikbaarheid van faciliteiten/mensen realistisch?
- Zijn de verwachte resultaten, fasering en tijdsplanning realistisch?

Kennisoverdracht en implementatie

Denk daarbij aan:
- goede mix van activiteiten voor kennisoverdracht en/of bestendiging;
- mate waarin de betrokkenheid van gebruikers en doelgroepen (inclusief patiëntenvertegenwoordigers) aandacht krijgt;
- is de implementatiestrategie duidelijk?

2. Samenvattend oordeel kwaliteit

Legenda: ZG (zeer goed), G (goed), V (voldoende), M (matig), O (onvoldoende)

2.1 Sterke punten kwaliteit: toelichting naar de aanvragers

Het voorstel bestaat waarschijnlijk uit meerdere werkpakketten. Maak in uw beoordeling per punt duidelijk op welk werkpakket uw commentaar betrekking heeft.

Benoem sterke punten per onderdeel:

1.1. Probleemstelling en doelstellingen

1.2. Plan van aanpak

1.3. Haalbaarheid

1.4. Kennisoverdracht en implementatie

De verschillende WP's zijn helder omschreven. WP1-3 lijken erg op elkaar. Voor WP2 staat de analyse beschreven, voor WP1 en WP3 niet. Is dat nog niet bepaald?
WP4 is compleet anders en onderverdeeld in twee sub-werkpakketten.
WP1-3 putten data uit bestaande databanken. WP4 put uit verschillende biobanken.
Tot zover is de studieopzet helder en duidelijk beschreven.

2.2 Zwakke punten kwaliteit: toelichting naar de aanvragers

Het voorstel bestaat waarschijnlijk uit meerdere werkpakketten. Maak in uw beoordeling per punt duidelijk op welk werkpakket uw commentaar betrekking heeft.

Benoem zwakke punten per onderdeel:

1.1. Probleemstelling en doelstellingen

1.2. Plan van aanpak

1.3. Haalbaarheid

1.4. Kennisoverdracht en implementatie

Voor WP1 wordt een interventie aangegeven na 10 dagen dexamethason. Dit gaat toch alleen over een record stratificatie en niet om een patientpopulatie splitsing? Zelfde vraag voor WP2-3.
Bij WP4 staat "Gene expression data are analyzed using supervised and unsupervised analytical approaches and machine learning algorithms". Welke en hoeveel variabelen zijn daarbij betrokken (hoeveel dimensies heeft de clusteranalyse) en wat is de rol van een MLA?
Omdat er geen power analyse is gemaakt moet ervoor gewaakt worden dat de studiepopulatie niet breder (variabelen) dan diep is (aantal patiënten per tak). Er wordt ook geen post hoc power analyse gemaakt. Is dat omdat het geen random sample is?
Het is een multicentre- multi database retrospectieve studie. Hoe wordt de data schoon gehouden. Is er een ervaren datamanagementgroep betrokken bij metadata vergelijkingen, plausibiliteitanalyses, missing data (missing-at-random en missing not-at-random analyse) en double detection?
Het is mogelijk dat één en dezelfde patiënt in WP1 2 en 3 zit als ik het goed begrijp. Dit maakt de onderzoekspopulaties onderling afhankelijk met allerlei statistische problemen van dien. Is er een protocol om dit te voorkomen of te analyseren?

2.3 Samenvattend oordeel kwaliteit

ZG	G	V	M	O
		X		

Geef een samenvattend oordeel over de kwaliteit van de aanvraag.

Zeer goed: Het voorstel adresseert de kwaliteitscriteria zeer goed, en ik heb eventueel een aantal suggesties voor kleine verbeteringen

Goed: Het voorstel adresseert de kwaliteitscriteria goed, maar een aantal verbeteringen zijn noodzakelijk

Voldoende: In het algemeen zijn de kwaliteitscriteria geadresseerd, maar er zijn significante tekortkomingen die gecorrigeerd dienen te worden

Matig: Het voorstel vertoont tekortkomingen met betrekking tot de kwaliteitscriteria, verbetering is noodzakelijk

Onvoldoende: Het voorstel vertoont ernstige tekortkomingen met betrekking tot de kwaliteitscriteria

3. Relevantiecriteria

Beantwoorden kennishiaat
Het onderzoek levert de urgent benodigde kennis om het in de kennisagenda COVID-19 beschreven kennishiaat te beantwoorden. Geef indien mogelijk aan welke werkpakketten het meest urgent en welke het minst urgent zijn voor het beantwoorden van de kennisvraag.

Impact.
Het onderzoek is van groot belang omdat het gedurende de pandemie, en de nasleep daarvan, impact kan hebben op de behandeling van COVID-19 patiënten; of bijdraagt aan het beter voorbereid zijn op een eventuele volgende pandemie.

Implementeerbare tussenresultaten.
De aanvraag moet overtuigend onderbouwen dat onderzoek op korte termijn leidt tot implementeerbare (tussen)resultaten (tenminste tijdens de pandemie en daarnaast ook voor de lange termijn).

Unieke
positie onderzoeksveld en toegevoegde waarde naast ander onderzoek, er wordt zoveel mogelijk gebruik gemaakt van lopend onderzoek en/of registraties en cohorten

Nieuw onderzoek
Het onderzoek wordt niet al (inter)nationaal uitgevoerd (aanvrager moet dit overtuigend onderbouwen), of de aanvrager moet overtuigend onderbouwen dat onderzoek in Nederland nodig is naast internationaal onderzoek.

Geschikte onderzoeksgroep
Er is een competente onderzoeksgroep/ consortium/ samenwerkingsverband beschikbaar om dit op korte termijn op te pakken. In het consortium is een goede balans tussen academische, STZ en perifere ziekenhuizen en waar van toepassing samenwerking met eerstelijnsorganisaties. Kennishiaat wordt zoveel mogelijk in samenhang opgepakt, door de zorgketen heen.

Voortbouwen op lopend onderzoek en bestaande cohorten en/of databestanden
In deze ronde is in principe alleen financiering beschikbaar voor projecten die voortbouwen op lopend onderzoek en/of gebruik maken, koppelen en doorontwikkelingen van bestaande cohorten en/of databestanden.

Draagvlak
Bij beroepsgroepen en zorginstellingen is voldoende geborgd

Patiëntenparticipatie
Patiëntenparticipatie in het consortium is vereist, als partner vanaf protocol ontwikkeling. Met participatie wordt concreet bedoeld het raadplegen, advies inwinnen, samenwerken en/of laten (mee)beslissen van betrokkenen.

Diversiteit
Aanvragers dienen in de opzet, uitvoering, analyse en rapportage van resultaten onderscheid te maken tussen mannen en vrouwen en andere relevante subgroepen op basis van diversiteit. Indien diversiteit niet relevant is voor het voorgestelde onderzoek, wordt dit voldoende onderbouwd?

4. Samenvattend oordeel relevantie

Legenda: ZR (Zeer relevant), R (Relevant), LR (Laag relevant)

4.1 Sterke punten relevantie: toelichting naar de aanvragers
Het voorstel bestaat waarschijnlijk uit meerdere werkpakketten. Maak in uw beoordeling per punt duidelijk op welk werkpakket uw commentaar betrekking heeft

Benoem sterke punten per onderdeel en benoem duidelijk over werkpakket het gaat:

3.1. Beantwoorden kennishiaat

3.2. Impact

3.3. Implementeerbare tussenresultaten

3.4. Unieke positie

3.5. Nieuw onderzoek

3.6. Geschikte onderzoeksgroep

3.7. Voortbouwen op lopend onderzoek en bestaande cohorten en/of databestanden

3.8. Draagvlak

3.9. Patientenparticipatie

3.10. Diversiteit

Zoals de aanvragers schrijven is het medicatieprotocol van levensbelang. Wat, wanneer en hoeveel. In retrospectief wordt geprobeerd om uit de ervaringen het best toepasbare protocol te herleiden, samen met de eventueel van belang zijnde patiënt parameters. Zo'n observationeel, niet gerandomiseerd onderzoek is vanwege allerlei verstorende factoren (ziekenhuis, periode van behandeling) een uitdaging. Toch is dit de enige beschikbare informatie en is een gerandomiseerde studie op dit moment niet haalbaar. Omdat er gebruik gemaakt wordt van bestaande data om onderzoek te doen naar fenomenen waar eigenlijk niks van bekend is, is dit onderzoek een eerste essentiële stap.

4.2 Zwakke punten relevantie: toelichting naar de aanvragers
Het voorstel bestaat waarschijnlijk uit meerdere werkpakketten. Maak in uw beoordeling per punt duidelijk op welk werkpakket uw commentaar betrekking heeft

Benoem zwakke punten per onderdeel:

3.1. Beantwoorden kennishiaat

3.2. Impact

3.3. Implementeerbare tussenresultaten

3.4. Unieke positie

3.5. Nieuw onderzoek

3.6. Geschikte onderzoeksgroep

3.7. Voortbouwen op lopend onderzoek en bestaande cohorten en/of databestanden

3.8. Draagvlak

3.9. Patientenparticipatie

3.10. Diversiteit

De onderzoeksgroep bevat veel medisch inhoudelijk deskundigen, maar het probleemgebied ligt vooral bij de data en metadata verzameling. Ik zou willen aanraden om de aanvraag voor een arts-onderzoeker/data engineer te vervangen door een ervaren data-engineer. Of deze expertise in te huren bij één van de data science groepen van de betrokken universiteiten

4.3 Samenvattend oordeel relevantie

ZR	R	LR
	X	

Geef een samenvattend relevantie oordeel

5. Begroting

Legenda: TH (Te hoog), R (reëel), TL (te laag)

5.1 Begroting

TH	R	TL
	X	

In hoeverre is het budget realistisch gezien de geplande activiteiten? Is de toelichting op de begroting voldoende helder?

Overweeg één arts-onderzoeker/data-engineer te vervangen door een ervaren data scientist
